# Association of weight loss strategies with all-cause and specific-cause mortality: a prospective cohort study

**DOI:** 10.1186/s12889-024-19472-z

**Published:** 2024-08-16

**Authors:** Zhiquan Diao, Yilin Zhu, Wenqi Huang, Huiyan Wen, Jiaxin Li, Jiamin Qiu, Yingying Niu, Haoyu Yan, Jianfeng Zhong, Xuerui Bai, Zhitong Xu, Xiaofeng Liang, Dan Liu

**Affiliations:** 1 Department of Public Health and Preventive Medicine, School of Medicine, Jinan University, Guangzhou, Guangdong 510632 China; 2 Jinan University-University of Birmingham Joint Institute, Jinan University, Guangzhou, China; 3 School of Mathematics, College of Engineering and Physical Sciences, University of Birmingham, Birmingham, UK; 4 Laboratory of Viral Pathogenesis & Infection Prevention and Control, Jinan University, Ministry of Education, Guangzhou, China; 5 Department of Epidemiology, School of Public Health, Southern Medical University, Guangzhou, 510515 Guangdong China

**Keywords:** Weight loss strategies, All-cause, Specific-cause, Mortality, Cohort study

## Abstract

**Background:**

The health effects of different weight loss strategies vary greatly, and the relationship between weight loss strategies, especially the combination of multiple strategies, and death is still unclear. We aimed to examine the associations of various numbers and combinations of weight loss strategies with all-cause and specific-cause mortality and to further evaluate the associations of different total weight loss volumes with mortality.

**Methods:**

Using data from NHANES (1999–2018) with 48,430 participants aged 20 and above, we collected fourteen self-reported weight loss strategies and identified five clusters using latent class analysis. Cox proportional hazards models were used to examine the association between the amounts and clusters of weight loss strategies and mortality.

**Results:**

During a median follow-up of 9.1 years of 48,430 participants, 7,539 deaths were recorded (including 1,941 CVDs and 1,714 cancer). Participants who adopted 2, 3–4, and ≥ 5 weight loss strategies had a lower risk of all-cause mortality, with HRs of 0.88 (95% CI, 0.81 to 0.97), 0.89 (95% CI, 0.81 to 0.96) and 0.71 (95% CI, 0.61 to 0.82). Regardless of weight loss or weight gain categories, there was a significant trend toward reduced mortality as the number of weight loss strategies increased (*P* trend < 0.05). Participants who adopted cluster-1 (four strategies), cluster-2 (five strategies) and cluster-3 (three strategies) had a significantly lower risk of all-cause mortality, with HRs of 0.71 (95% CI, 0.60 to 0.84), 0.70 (95% CI, 0.55 to 0.89) and 0.81 (95% CI, 0.70 to 0.94). Among them, cluster-1 and cluster-2 are both characterized by eating less food, exercising, drinking plenty of water, lowering calories and eating less fat. Conversely, cluster-4 (five strategies) and cluster-5 (four strategies) had marginally significant effects, and they both had actual higher total energy intakes. Similar associations were observed for CVDs and cancer mortality.

**Conclusions:**

Employing two or more weight loss strategies was associated with a lower risk of death, even among those who gained weight. Eating less food, exercising, drinking plenty of water, lowering calories and eating less fat is a better combination of strategies. On this basis, limiting the actual intake of total energy is necessary.

**Supplementary Information:**

The online version contains supplementary material available at 10.1186/s12889-024-19472-z.

## Background

The prevalence of obesity has increased worldwide, bringing enormous health challenges [1]. According to the World Obesity Federation, by 2035, 187 million (47%) of American adult men and 208 million (49%) of American adult women will have obesity [2]. Weight management is a key focus in preventing and controlling obesity, including changes in of lifestyle factors (e.g., dietary habits and physical activity), the use of medications, and, in some cases, surgical recommendations [3]. Several studies have demonstrated that following weight loss strategies that limiting energy intake and being physically active can significantly reduce cardiovascular diseases (CVDs) risk factors and CVDs risk [4, 5]. A review including 122 randomized clinical trials (RCTs) and 2 observational studies found that behavioral and pharmacotherapy weight loss interventions among individuals who have obesity may lead to a small decrease in mortality risk [6]. Willis et al. found that an increased frequency of intentional weight loss in middle age was associated with a lower risk of death [7]. To our knowledge, most of the current studies focus on the health effects of single or dual weight loss strategies, whereas in reality, people often tend to use several weight loss strategies at once [8]. Therefore, the relationship between the number of weight loss strategies used and long-term health outcomes remains unclear. Meanwhile, the relationship of the combination of different weight loss strategies on health outcomes remains unclear. In addition, there is little research to clarify the effect of different total volumes of weight loss on health outcomes. Thus, more studies investigating the long-term effects of multiple weight loss strategies and combinations of different weight loss strategies are needed.

In this study, nationally representative data from 10 consecutive surveys of the NHANES (1999–2000 to 2017–2018) were used, and we aimed to examine the associations of amounts and combinations of weight loss strategies with all-cause and specific-cause mortality and to further evaluate the associations of different total weight loss volumes with mortality.

## Methods

### Study design and participants

NHANES is a national program designed to assess the health and nutritional status of both adults and children in the United States [9], and detailed information regarding the sampling design and data collection for NHANES can be found on the official website [10]. Briefly, NHANES employs a stratified, multistage, probability sampling approach to collect nationally representative health-related data on the U.S. population. Comprehensive data collection methods were utilized, including completion of a household screener, a household interview, physical examinations and provision of biological samples. It has been conducted since 1999, with a survey cycle of every two years, continuously collecting demographic data, socioeconomic, dietary nutrition and health-related outcomes of the participants. NHANES was approved by the Ethics Review Committee of the National Center for Health Statistics. All participants provided informed consent.

In this analysis, we included 101,316 participants from 10 consecutive surveys of NHANES (1999–2000 to 2017–2018). We excluded participants who were ineligible for follow-up (*n* = 42,252) or younger than 20 years of age at baseline (*n* = 4,119), who had incomplete information on weight loss strategies (*n* = 60), who were underweight (i.e., baseline body mass index (BMI) < 18.5 kg/m^2^, *n* = 837) or had missing baseline BMI (*n* = 3,696), who had a positive lab pregnancy test or self-reported pregnancy or who were unable to determine pregnancy at the time of examination (*n* = 1,922). Ultimately, a total of 48,430 participants with a baseline age of 20 years and above, nonpregnant, and BMI ≥ 18.5 kg/m^2^ were included in the analysis. Figure S1 shows the inclusion screening process of the study participants.

### Assessment of weight loss strategies

In the household interview, the participants were asked “During the past 12 months, have you tried to lose weight?” If the participants answered “yes”, then they were further asked “How did you try to lose weight?”. A variety of weight loss strategy options were provided. From 1999 to 2000 to 2017–2018, the NHANES provided a list of 14 to 21 options. The following 14 options were included in all surveys: (1) ate less food, (2) lowered calories, (3) ate less fat, (4) exercised, (5) skipped meals, (6) consumed diet foods or products, (7) used a liquid diet formula, (8) joined a weight loss program, (9) took prescription diet pills, (10) took nonprescription diet pills, (11) took laxatives or vomiting, (12) drank a lot of water, (13) followed a special diet, and (14) other strategies. Starting in 2005, the NHANES survey added five additional options: (1) reduced carbohydrate intake; (2) began or resumed a smoking habit; (3) increased intake of fruits, vegetables, and salads; (4) altered eating habits (e.g., no food consumption late at night); (5) reduced intake of sugar, candy, and sweets; starting in 2009, an additional option (6) reduced consumption of junk food or fast food was provided; and starting in 2013, an additional option (7) had weight loss surgery to lose weight was provided. The strategies were not mutually exclusive, participants could choose one or more of them, and information on weight loss strategies was reported only once for each participant.

We included 14 weight loss strategies that were presented in all surveys in our main analysis. First, we counted the various numbers and percentages of participants who took from 0 to 14 cumulative weight loss strategies and the incidence of mortality. Based on trends between the number of weight loss strategies and mortality, participants were divided into five groups: 0 (no weight loss attempt), 1, 2, 3–4 and ≥ 5 (5 or more weight loss strategies) (Table S1). Then, to distinguish the association between various combinations of weight loss strategies and death, we used latent class analysis to cluster 14 weight loss strategies into 5 clusters based on the stable low values of statistical fit index (BIC), the peak values of class separation quality (entropy), and the interpretability of the clusters (Figure S2).

### Ascertainment of mortality

Deaths were identified by linking NHANES participants to death certificate records from the National Death Index (NDI) and tracked through December 31, 2019. We classified causes of death according to International Statistical Classification of Diseases, 10th revision (ICD-10) codes. The primary outcomes of our study were all-cause mortality, CVD mortality (codes I00-I09, I11, I13 and I20-I51) and cancer mortality (codes C00-C97).

### Assessments of covariates

Potential confounders were identified from the literature [11, 12]and incorporated into a directed acyclic graph that was used to guide the modeling strategy (Figure S3). Information on covariates was obtained from the baseline information, including gender, age, race/ethnicity, education level, marital status, family income poverty ratio, weight change since last year, waist circumference, BMI, self-considered weight status, smoking status, drinking status, physical activity, total energy intakes, family history of prediabetes or diabetes and heart diseases, baseline history of diabetes, hypertension, high cholesterol, coronary heart disease and cancer, and history of medication for diabetes, hypertension and high cholesterol.

Data on BMI (kg/m^2^) and waist circumference (cm) were collected in the Mobile Examination Center (MEC) by trained health technicians. According to the classification standard of BMI by the National Institute of Health (NIH) and the World Health Organization (WHO) [13], we divided BMI into three groups: normal weight (18.5 to < 24.9 kg/m^2^), overweight (25.0 to < 29.9 kg/m^2^), and obesity (≥ 30.0 kg/m^2^). The family income poverty ratio was calculated by dividing family income by the poverty guidelines, specific to the appropriate year and participant’s state. We divided the ratio into three levels: low-income level (0 to ≤ 1.0), middle-income level (1.1 to ≤ 3.0), and high-income level (> 3.0) [14]. Physical activity was assessed by investigating at least 10 min of moderate or vigorous activity in the past 30 days that resulted in sweating or increased breathing or heart rate, such as brisk walking, cycling, golfing, and dancing. Total energy intakes (kcal/d) was collected in person at the MEC in one 24-hour dietary recall interview. History of diseases was obtained by asking whether they had been informed of a disease by a doctor or health professional, and medication use was collected by self-report questionnaire or verbal interviews. Weight change since last year (pounds) was estimated as the difference value between current self-reported weight and self-reported weight one year ago. Details of the covariates assessed in NHANES are provided in Table S2.

### Statistical analysis

Detailed information on missing covariates is presented in Table S3. To account for these missing values, the multiple imputation by chained equations (MICE) method was used, and 10 datasets were created through this imputation process. All variables, including the outcomes, were included in the multiple imputation model, ensuring a comprehensive imputation of missing values. Baseline characteristics are presented as the mean (standard deviation [SD]) for continuous variables and number (percentage [%]) for categorical variables.

We defined baseline as the time at which participants conducted their interviews. We calculated person-years from baseline to death, loss to follow-up, or December 31, 2019, whichever occurred first. Details of the description have been published elsewhere [15]. We compared baseline characteristics of participants who used different numbers of weight loss strategies by using the Rao-Scott χ^2^ test for categorical variables and analysis of variance for continuous variables. The proportional hazards assumption was tested using Schoenfeld residuals. We used the Cox proportional hazards model to calculate hazard ratios (HRs) and 95% confidence intervals (CIs) for the association of five groups of cumulative weight loss strategies (0, 1, 2, 3–4, ≥ 5) with all-cause and specific-cause mortality. Then, we used the latent class analysis (LCA) [16–18] clustering algorithm to cluster and combine 14 weight loss strategies into 5 clusters to explore the relationship between the combinations of two or more weight loss strategies and mortality after removing participants who used only one weight loss strategy.

We constructed two models to estimate associations. Model 1 adjusted for baseline gender (male or female) and age (years, continuous). Model 2 further adjusted for race/ethnicity (Hispanic, or non-Hispanic), education level (less than high school, high school or equivalent, or college or above), marital status (married, widowed, divorced, separated, or never married), family income poverty ratio (ratio, continuous), weight change since last year (pounds, continuous), waist circumference (cm, continuous), BMI (normal, overweight, or obesity), self-considered weight status (underweight, about the right weight, or overweight), smoking status (never, past, or current), drinking status (never, past or current), physical activity (yes or no), total energy intakes (kcal/d, continuous), family history of prediabetes or diabetes and heart diseases (yes or no), baseline history of diabetes, hypertension, high cholesterol, coronary heart disease and cancer (yes or no), and history of medication for diabetes, hypertension and high cholesterol (yes or no). We also calculated linear trends with cumulative weight loss strategies as continuous variables.

Using cumulative weight loss strategies as a continuous variable, we performed stratified analyses to explore the relationship between weight loss strategies and all-cause mortality in subgroups of gender (male or female), age (20 to ≤ 44, 45 to ≤ 59 or ≥ 60), education level (less than high school, high school or equivalent, or college or above), family income poverty ratio (> 3.0 or ≤ 3.0), smoking status (never, past, or current), drinking status (never, past or current), BMI (18.5 to < 24.9, 25.0 to < 29.9, or ≥ 30.0 kg/m^2^) and physical activity (yes or no), and the interaction effects were estimated.

We performed a series of sensitivity analyses to examine the robustness of the results. First, we excluded participants with missing covariates, restricting the analysis to complete cases. Second, to minimize the reverse causality bias, participants who had CVDs and cancer at baseline, who died within the first three years of follow-up or who did not report intentional weight loss but lost more than 10 pounds were excluded from the analysis. Third, we excluded participants with normal weight, and the analysis was restricted to participants with overweight or obesity (BMI ≥ 25.0 kg/m^2^) to reduce the health effects among normal weight people. Fourth, we used waist circumference to define obesity (men ≥ 102 cm, women ≥ 88 cm) [19], limiting the study to participants with abdominal obesity to analyze the association of weight loss strategies with mortality. Fifth, to reduce the impact of the limitations of the NHANES questionnaire settings on the results, we removed participants who adopted “other strategies” from the 14 weight loss strategies or included participants who additionally adopted any of the newly added 7 weight loss strategies since 2005 as “other strategies” for analysis. Finally, we further adjusted the survey rounds and country of birth to reduce the effect of the birth cohort.

All statistical analyses were performed using R statistical software (version 4.3.0). We used the R package “mice” to perform efficient multiple imputation of all missing values [20]. All statistical tests were two-sided, and the test level α = 0.05, *p* < 0.05 was considered statistically significant.

## Results

### Baseline characteristics

As Table 1 shows, among 48,430 participants, the mean age at baseline was 50.3 (SD 17.9) years, 49.8% were female, and 12,469 (25.7%) were Hispanic. Among them, 28,365 (58.6%) participants did not try to lose weight, 4,616 (9.5%) adopted 1 weight loss strategy, 5,043 (10.4%) adopted 2 weight loss strategies, 6,881 (14.2%) adopted 3 or 4 weight loss strategies, and 3,525 (7.3%) participants adopted 5 or more strategies. Compared with participants who did not try to lose weight, those who followed weight loss strategies were more likely to be female, younger, with obesity being present, self-perceived as overweight, nonsmokers, have lower energy intake and be physically active. Simultaneously, they were more likely to report a family history of prediabetes or diabetes and heart diseases, a baseline history of diabetes, hypertension and high cholesterol, and a history of medication, but less likely to report a history of cancer. Participants who adopted more weight loss strategies had higher education levels and household incomes, larger waist circumferences and BMIs, and more weight loss since last year. Participants who adopted 5 or more weight loss strategies had the largest proportion of obesity and weight loss and the lowest proportion of coronary heart disease.

**Table 1:** Baseline characteristics of participants in NHANES 1999–2018 according to the number of weight loss strategies

Characteristics	Total	The number of weight loss strategies					*P* value ‡
		0	1	2	3–4	≥ 5	
**Participants**,** n (%)**	48,430	28,365 (58.6)	4616 (9.5)	5043 (10.4)	6881 (14.2)	3525 (7.3)	< 0.001
**Female**,** n (%)**	24,142 (49.8)	12,369 (43.6)	2493 (54.0)	2844 (56.4)	4090 (59.4)	2346 (66.6)	< 0.001
**Mean age (SD)**,** y**	50.3 (17.9)	51.7 (18.7)	50.8 (17.1)	49.2 (16.8)	47.9 (16.5)	44.7 (15.3)	< 0.001
**Ethnicity (%)**							< 0.001
Hispanic	12,469 (25.7)	7285 (25.7)	1283 (27.8)	1375 (27.3)	1766 (25.7)	760 (21.6)	
Non-Hispanic	35,961 (74.3)	21,080 (74.3)	3333 (72.2)	3668 (72.7)	5115 (74.3)	2765 (78.4)	
**Education**,** n (%)**							< 0.001
Less than high school	13,148 (27.1)	9048 (31.9)	1267 (27.4)	1135 (22.5)	1284 (18.7)	414 (11.7)	
High school or equivalent	11,268 (23.3)	6841 (24.1)	1114 (24.1)	1157 (22.9)	1432 (20.8)	724 (20.5)	
College or above	24,014 (49.6)	12,476 (44.0)	2235 (48.4)	2751 (54.6)	4165 (60.5)	2387 (67.7)	
**Marital status**,** n (%)**							< 0.001
Married	25,594 (52.8)	14,750 (52.0)	2545 (55.1)	2789 (55.3)	3684 (53.5)	1826 (51.8)	
Widowed	4234 (8.7)	2948 (10.4)	349 (7.6)	352 (7.0)	431 (6.3)	154 (4.4)	
Divorced	5081 (10.5)	2798 (9.9)	524 (11.4)	546 (10.8)	789 (11.5)	424 (12.0)	
Separated	1644 (3.4)	951 (3.4)	150 (3.2)	194 (3.8)	223 (3.2)	126 (3.6)	
Never married	11,877 (24.5)	6918 (24.4)	1048 (22.7)	1162 (23.0)	1754 (25.5)	995 (28.2)	
**Mean family income poverty ratio (SD)**	2.5 (1.6)	2.4 (1.6)	2.5 (1.6)	2.7 (1.6)	2.8 (1.6)	3.0 (1.6)	< 0.001
**Mean weight change since last year (SD)**,** pounds**	-0.3 (16.9)	0.9 (13.6)	-1.3 (18.5)	-2.3 (20.4)	-2.4 (20.2)	-2.7 (23.9)	< 0.001
**Mean waist circumference (SD)**,** cm**	99.3 (15.8)	95.7 (14.5)	104.0 (15.7)	104.3 (16.1)	104.4 (16.1)	104.9 (16.8)	< 0.001
**Mean body mass index (SD)**,** kg/m**^**2**^	29.1 (6.7)	27.3 (5.8)	31.2 (6.7)	31.5 (6.9)	31.8 (6.9)	32.5 (7.4)	< 0.001
Normal, n (%)	13,850 (28.6)	11,138 (39.3)	678 (14.7)	701 (13.9)	901 (13.1)	432 (12.3)	
Overweight, n (%)	16,659 (34.4)	9988 (35.2)	1615 (35.0)	1745 (34.6)	2247 (32.7)	1064 (30.2)	
Obesity, n (%)	17,921 (37.0)	7239 (25.5)	2323 (50.3)	2597 (51.5)	3733 (54.2)	2029 (57.5)	
**Self-considered weight status**,** n (%)**							< 0.001
Underweight	2189 (4.5)	1997 (7.0)	43 (0.9)	48 (1.0)	65 (0.9)	36 (1.0)	
About the right weight	20,135 (41.6)	15,869 (55.9)	1145 (24.8)	1144 (22.7)	1412 (20.5)	565 (16.0)	
Overweight	26,106 (53.9)	10,499 (37.0)	3428 (74.3)	3851 (76.4)	5404 (78.5)	2924 (83.0)	
**Smoking status**,** n (%)**							< 0.001
Never	26,193 (54.1)	14,558 (51.3)	2629 (57.0)	2888 (57.3)	4008 (58.2)	2110 (59.9)	
Past	12,117 (25.0)	6919 (24.4)	1203 (26.1)	1326 (26.3)	1810 (26.3)	859 (24.4)	
Current smoker	10,120 (20.9)	6888 (24.3)	784 (17.0)	829 (16.4)	1063 (15.4)	556 (15.8)	
**Drinking status**,** n (%)**							< 0.001
Never	13,279 (27.4)	7864 (27.7)	1358 (29.4)	1410 (28.0)	1808 (26.3)	839 (23.8)	
Past or current	35,151 (72.6)	20,501 (72.3)	3258 (70.6)	3633 (72.0)	5073 (73.7)	2686 (76.2)	
**Physical activity**,** n (%)**	26,002 (53.7)	13,469 (47.5)	2334 (50.6)	3032 (60.1)	4479 (65.1)	2688 (76.3)	< 0.001
**Mean total energy intakes (SD)**,** kcal/d**	2101.6 (1004.5)	2160.8 (1052.3)	2029.9 (961.1)	2006.9 (933.2)	2022.7 (893.1)	2008.2 (933.7)	< 0.001
**Diseases of family history**,** n (%)**							< 0.001
Prediabetes or diabetes	21,448 (44.3)	11,570 (40.8)	2131 (46.2)	2424 (48.1)	3452 (50.2)	1871 (53.1)	
Heart diseases	6017 (12.4)	3252 (11.5)	615 (13.3)	641 (12.7)	958 (13.9)	551 (15.6)	
**Diseases at baseline**,** n (%)**							< 0.001
Diabetes	6041 (12.5)	3105 (10.9)	717 (15.5)	763 (15.1)	1023 (14.9)	433 (12.3)	
Hypertension	17,241 (35.6)	9394 (33.1)	1883 (40.8)	2014 (39.9)	2653 (38.6)	1297 (36.8)	
High cholesterol	15,648 (32.3)	8278 (29.2)	1658 (35.9)	1835 (36.4)	2573 (37.4)	1304 (37.0)	
Coronary heart disease	2080 (4.3)	1301 (4.6)	184 (4.0)	234 (4.6)	266 (3.9)	95 (2.7)	
Cancer	4586 (9.5)	2855 (10.1)	432 (9.4)	443 (8.8)	576 (8.4)	280 (7.9)	
**Use of prescribed medicine**,** n (%)**							< 0.001
Diabetes	4661 (9.6)	2306 (8.1)	582 (12.6)	630 (12.5)	792 (11.5)	351 (10.0)	
Hypertension	13,064 (27.0)	7167 (25.3)	1437 (31.1)	1523 (30.2)	1987 (28.9)	950 (27.0)	
High cholesterol	8452 (17.5)	4529 (16.0)	946 (20.5)	1016 (20.1)	1358 (19.7)	603 (17.1)	

‡ For categorical variables, *P* value was calculated by Rao-Scott χ^2^ test. For continuous variables, analysis of variance was used to calculate *P* value

### Associations of the number of weight loss strategies with mortality

During a median follow-up of 9.1 years (interquartile range, 4.8 to 13.7 years) and a total of 461,257 person-years, 7,539 all-cause mortalities were recorded (including 1,941 CVDs and 1,714 cancers). Table 2 shows the relationship between cumulative weight loss strategies and risk of death. Compared with participants who did not try to lose weight, participants who adopted five or more weight loss strategies had the lowest mortality rate (5.80 vs. 19.83 per 1,000 person years). Participants who adopted 2, 3–4, 5 or more weight loss strategies had a lower risk of all-cause mortality, with HRs of 0.88 (95% CI, 0.81 to 0.97), 0.89 (95% CI, 0.81 to 0.96) and 0.71 (95% CI, 0.61 to 0.82), respectively. For each weight loss strategy increase, the risk of all-cause mortality decreased by 6% (*P* trend < 0.00, HR = 0.94, 95% CI: 0.92 to 0.96). Similar associations were observed for CVDs and cancer mortality. For each additional weight loss strategy, the risk of CVDs and cancer death was reduced by 5% (*P* trend = 0.04, HR = 0.95, 95% CI: 0.91 to 1.00) and 7% (*P* trend = 0.00, HR = 0.93, 95% CI: 0.89 to 0.98), respectively. As more strategies are adopted, this trend of reduced risk of death is mainly related to the large proportion of “ate less food” and “exercised”, and the continuous increase of “lowered calories”, “ate less fat” and “drank a lot of water” (Figure S4).

**Table 2:** Hazard ratios (95% CIs) of mortality with the number of weight loss strategies in NHANES 1999–2018

Mortality	The number of weight loss strategies					*P* value for trend	Per 1-strategy increment
	0	1	2	3–4	≥ 5		
All cause							
Incident rate‡	19.83	14.91	12.32	10.94	5.80		
Cases/N	5418/28,365	647/4616	576/5043	703/6881	195/3525		
Model1*	1.00 (reference)	0.89 (0.82 to 0.96)	0.81 (0.75 to 0.89)	0.80 (0.74 to 0.87)	0.60 (0.52 to 0.69)	< 0.00	0.91 (0.89 to 0.93)
Model2†	1.00 (reference)	0.93 (0.86 to 1.01)	0.88 (0.81 to 0.97)	0.89 (0.81 to 0.96)	0.71 (0.61 to 0.82)	< 0.00	0.94 (0.92 to 0.96)
**CVD**							
Incident rate‡	5.02	4.12	3.34	3.08	1.07		
Cases/N	1372/28,365	179/4616	156/5043	198/6881	36/3525		
Model1*	1.00 (reference)	1.00 (0.86 to 1.17)	0.91 (0.77 to 1.08)	0.96 (0.82 to 1.11)	0.49 (0.35 to 0.69)	0.00	0.94 (0.90 to 0.98)
Model2†	1.00 (reference)	1.00 (0.85 to 1.17)	0.94 (0.79 to 1.12)	0.99 (0.85 to 1.17)	0.54 (0.39 to 0.76)	0.04	0.95 (0.91 to 1.00)
**Cancer**							
Incident rate‡	4.43	3.43	2.99	2.62	1.40		
Cases/N	1210/28,365	149/4616	140/5043	168/6881	47/3525		
Model1*	1.00 (reference)	0.89 (0.75 to 1.06)	0.85 (0.72 to 1.02)	0.83 (0.70 to 0.97)	0.60 (0.44 to 0.80)	< 0.00	0.92 (0.88 to 0.96)
Model2†	1.00 (reference)	0.91 (0.76 to 1.08)	0.89 (0.74 to 1.07)	0.87 (0.73 to 1.03)	0.65 (0.48 to 0.88)	0.00	0.93 (0.89 to 0.98)

* Model 1 was adjusted for baseline age (years, continuous) and gender (male or female)

† Model 2 was additionally adjusted by race/ethnicity (Hispanic, or non-Hispanic), education level (less than high school, high school or equivalent, or college or above), marital status (married, widowed, divorced, separated, or never married), family income poverty ratio (ratio, continuous), weight change since last year (pounds, continuous), waist circumference (cm, continuous), BMI (normal, overweight, or obesity), self-considered weight status (underweight, about the right weight, or overweight), smoking status (never, past, or current smoker), drinking status (never, past or current), physical activity (yes or no), total energy intakes (kcal/d, continuous), family history of prediabetes or diabetes and heart diseases (yes or no), baseline history of diabetes, hypertension, high cholesterol, coronary heart disease and cancer (yes or no), and history of medication for diabetes, hypertension and high cholesterol (yes or no)

‡ Incident rate per 1,000 person years

**Fig. 1:**
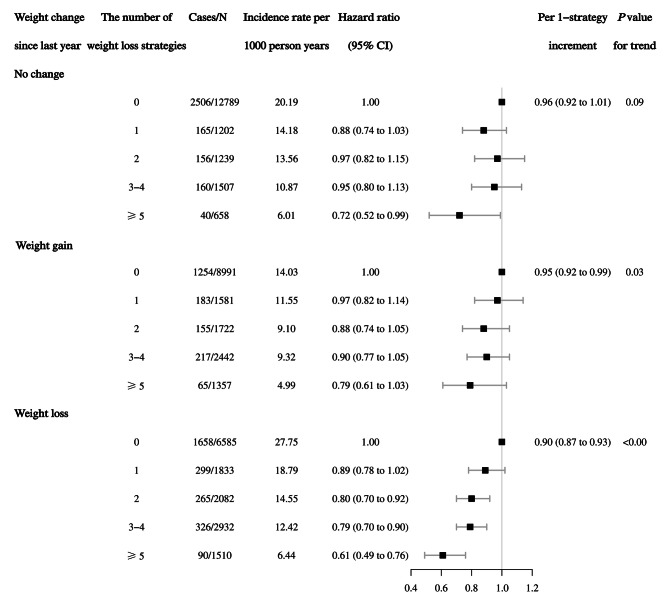
Association between weight loss strategies and risk of all-cause mortality stratified by weight change since last year. The hazard ratios of 95% CI taking into account for competing death risk were adjusted for baseline age (years, continuous), gender (male or female), race/ethnicity (Hispanic, or non-Hispanic), education level (less than high school, high school or equivalent, or college or above), marital status (married, widowed, divorced, separated, or never married), family income poverty ratio (ratio, continuous), weight change since last year (pounds, continuous), waist circumference (cm, continuous), BMI (normal, overweight, or obesity), self-considered weight status (underweight, about the right weight, or overweight), smoking status (never, past, or current smoker), drinking status (never, past or current), physical activity (yes or no), total energy intakes (kcal/d, continuous), family history of prediabetes or diabetes and heart diseases (yes or no), baseline history of diabetes, hypertension, high cholesterol, coronary heart disease and cancer (yes or no), and history of medication for diabetes, hypertension and high cholesterol (yes or no). The strata variable was not included in the model when stratifying by itself

To explore whether the observed associations were driven by the total volume of weight loss, we jointly classified categories of weight change since last year (no change, weight gain, and weight loss) and cumulative weight loss strategies (Fig. 1). Among weight loss categories, there was a significant trend towards reduced mortality with an increased number of weight loss strategies (*P* trend < 0.00) with an HR of 0.90 (95% CI, 0.87 to 0.93). Among the weight gain and no change categories, there was a marginally significant trend toward reduced mortality (*P* trend = 0.03 and 0.09) with HRs of 0.95 (95% CI, 0.92 to 0.99) and 0.96 (95% CI, 0.92 to 1.01), respectively.

### Associations of the combination of weight loss strategies with mortality

Based on the Bayesian Information Criterion (BIC), entropy and class separation quality, we clustered the 14 weight loss strategies into 5 clusters after removing 4,616 participants who used only one weight loss strategy. Participants in cluster-1 mainly adopted the four key weight loss strategies: “exercised”, “drank a lot of water”, “lowered calories”, and “ate less fat”. Participants in cluster-2 predominantly followed the same four strategies as “cluster-1”, with the addition of “ate less food”. Cluster-3 was primarily characterized by three strategies: “exercised”, “ate less food” and “drank a lot of water”. The main strategies of cluster-4 are the same as those of cluster-2. Cluster-5 was defined by the adoption of three strategies from cluster-3, along with the practice of “skipped meals”. Cluster-4 and − 5 had higher total energy intake (Table S4).

Compared with participants who did not try to lose weight, participants who adopted cluster-1 to cluster-3 had a significantly lower risk of all-cause mortality, with HRs of 0.71 (95% CI, 0.60 to 0.84), 0.70 (95% CI, 0.55 to 0.89) and 0.81 (95% CI, 0.70 to 0.94); while cluster-4 and cluster-5 had marginally significant effects, with HRs of 0.92 (95% CI, 0.85 to 1.00) and 0.88 (95% CI, 0.77 to 1.00), respectively. Only cluster-2 was significantly associated with lower risk of CVDs and cancer death (Table 3).

**Table 3:** Hazard ratios (95% CIs) of mortality with weight loss strategies clustered by latent class analysis in NHANES 1999–2018

Mortality	No weight loss attempt	The combination of weight loss strategies				
		Cluster-1	Cluster-2	Cluster-3	Cluster-4	Cluster-5
All cause						
Incident rate‡	19.83	5.57	5.15	8.99	14.24	9.62
Cases/N	5418/28,365	145/2914	73/1416	177/2177	814/5910	265/3032
Model1*	1.00 (reference)	0.56 (0.47 to 0.66)	0.59 (0.47 to 0.74)	0.69 (0.59 to 0.80)	0.85 (0.79 to 0.92)	0.85 (0.75 to 0.96)
Model2†	1.00 (reference)	0.71 (0.60 to 0.84)	0.70 (0.55 to 0.89)	0.81 (0.70 to 0.94)	0.92 (0.85 to 1.00)	0.88 (0.77 to 1.00)
**CVD**						
Incident rate‡	5.02	1.42	0.49	2.44	3.92	2.69
Cases/N	1372/28,365	37/2914	7/1416	48/2177	224/5910	74/3032
Model1*	1.00 (reference)	0.61 (0.44 to 0.85)	0.25 (0.12 to 0.53)	0.78 (0.58 to 1.04)	0.97 (0.84 to 1.12)	1.00 (0.79 to 1.26)
Model2†	1.00 (reference)	0.76 (0.55 to 1.06)	0.28 (0.13 to 0.58)	0.88 (0.65 to 1.18)	0.99 (0.85 to 1.15)	0.99 (0.78 to 1.26)
**Cancer**						
Incident rate‡	4.43	1.65	1.13	2.03	3.34	2.36
Cases/N	1210/28,365	43/2914	16/1416	40/2177	191/5910	65/3032
Model1*	1.00 (reference)	0.68 (0.50 to 0.93)	0.54 (0.33 to 0.88)	0.65 (0.47 to 0.89)	0.88 (0.75 to 1.02)	0.88 (0.68 to 1.13)
Model2†	1.00 (reference)	0.81 (0.59 to 1.11)	0.57 (0.35 to 0.94)	0.73 (0.53 to 1.01)	0.91 (0.77 to 1.07)	0.83 (0.64 to 1.08)

* Model 1 was adjusted for baseline age (years, continuous) and gender (male or female)

† Model 2 was additionally adjusted by race/ethnicity (Hispanic, or non-Hispanic), education level (less than high school, high school or equivalent, or college or above), marital status (married, widowed, divorced, separated, or never married), family income poverty ratio (ratio, continuous), weight change since last year (pounds, continuous), waist circumference (cm, continuous), BMI (normal, overweight, or obesity), self-considered weight status (underweight, about the right weight, or overweight), smoking status (never, past, or current smoker), drinking status (never, past or current), physical activity (yes or no), total energy intakes (kcal/d, continuous), family history of prediabetes or diabetes and heart diseases (yes or no), baseline history of diabetes, hypertension, high cholesterol, coronary heart disease and cancer (yes or no), and history of medication for diabetes, hypertension and high cholesterol (yes or no)

‡ Incident rate per 1,000 person years

### Subgroups and sensitivity analyses

**Fig. 2:**
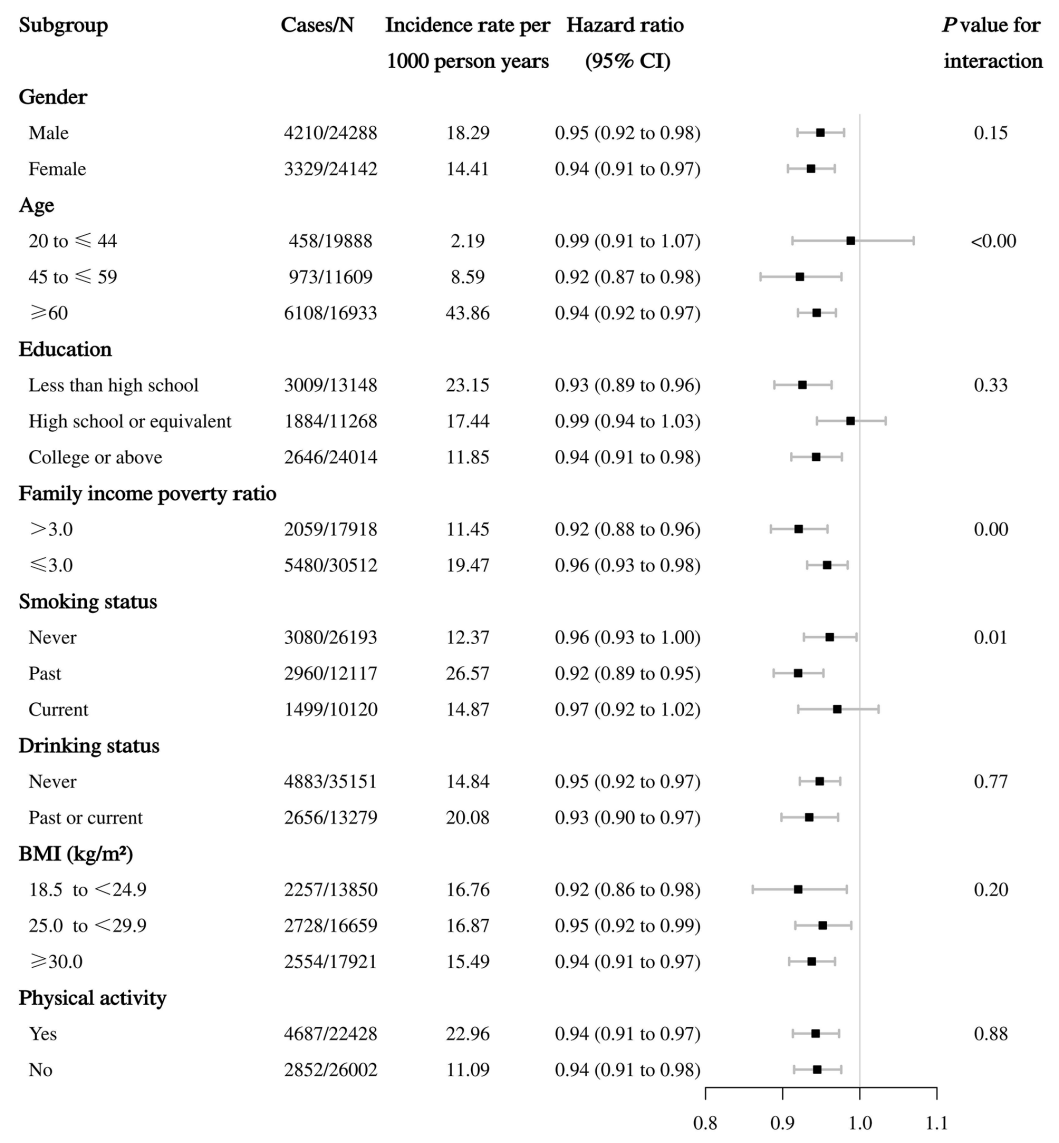
Association between weight loss strategies and risk of all-cause mortality stratified by subgroups. The hazard ratios of 95% CI taking into account for competing death risk were adjusted for baseline age (years, continuous), gender (male or female), race/ethnicity (Hispanic, or non-Hispanic), education level (less than high school, high school or equivalent, or college or above), marital status (married, widowed, divorced, separated, or never married), family income poverty ratio (ratio, continuous), weight change since last year (pounds, continuous), waist circumference (cm, continuous), BMI (normal, overweight, or obesity), self-considered weight status (underweight, about the right weight, or overweight), smoking status (never, past, or current smoker), drinking status (never, past or current), physical activity (yes or no), total energy intakes (kcal/d, continuous), family history of prediabetes or diabetes and heart diseases (yes or no), baseline history of diabetes, hypertension, high cholesterol, coronary heart disease and cancer (yes or no), and history of medication for diabetes, hypertension and high cholesterol (yes or no). The strata variable was not included in the model when stratifying by itself

Consistent findings were observed among participants with cumulative weight loss strategies (continuous) and risk of all-cause mortality when stratified by gender, age, education, family income poverty ratio, smoking status, drinking status, BMI categories, and physical activity. Notably, similar associations were found between cumulative weight loss strategies and all-cause mortality when stratified by BMI categories (*P* interaction = 0.20). Among participants aged 45–59 years, larger family income poverty ratio and past smokers, a stronger association was found between weight loss strategies and all-cause mortality (*P* interaction < 0.00) (Fig. 2).

In sensitivity analyses (Table S5-S13), none of which resulted in substantial changes, and the association of cumulative weight loss strategies with risk of death was consistent. However, there was a slightly attenuated linear trend effect when excluding participants who experienced an outcome event within the first three years of follow-up, or those with coronary heart disease and cancer history.

## Discussion

In this large prospective cohort study of nationally representative US adults from NHANES (1999–2018), we observed that participants taking at least two weight loss strategies were associated with a reduced risk of all-cause mortality, whereas taking five or more strategies reduced the largest risk of death. Associations were also noted for deaths caused by CVDs and cancer. Notably, this inverse association was observed even among those who gained weight since last year, suggesting the benefit of adopting multiple strategies in the weight loss process even if weight is slightly gained during the time. Furthermore, for the combined application of multiple strategies, we found that eating less food, exercising, drinking plenty of water, lowering calories and eating less fat is a better combination of strategies. On this basis, limiting the actual intake of calories is necessary. These findings are of high clinical and public health importance.

### Comparison with other studies

Most of the current research concerns the health effects of people adopting single or dual weight loss strategies [21–30]. A recent meta-analysis determined the worldwide prevalence of various weight loss and weight maintenance strategies [31]. An RCT study showed that combined behavior therapy yielded significantly greater weight loss than behavior therapy alone [32]. While, there is also evidence that engaging in a greater number of weight loss methods and attempts may decrease the likelihood of weight loss and maintenance within 1- and 2-year periods among individuals with obesity as well as increase the risk of weight gain [33]. Previous studies explored the association between attempted weight loss and all-cause mortality [7, 34]. However, the relationship of the cumulative number of weight loss strategies on long-term health outcomes remains unclear. In our study, we found that participants who adopted more weight loss strategies had a lower risk of all-cause mortality. Surprisingly, the reduced risk of mortality persisted even when considering cases where individuals had gained weight during the weight loss process. As indicated by previous studies, individuals with moderate or adequate health literacy tended to report a higher number of weight loss methods [35]. This observation implies that those employing a greater number of strategies may be more inclined toward adopting healthier behaviors. This may explain why people who employ multiple weight-loss strategies experience long-term health benefits even when they gain weight.

Public health guidelines always recommend a combination of reduced caloric intake and increased physical activity, along with additional behavioral changes, as an effective way to achieve long-term weight loss and health outcomes [36]. While combined weight loss strategies are commonly utilized in real-world settings [8], it is important to note that the optimal combination of these strategies for achieving the most effective results remains unknown. In our study, we found that participants who adopted weight loss strategies named as cluster-1 (four strategies), cluster-2 (five strategies) and cluster-3 (three strategies) had a significantly lower risk of all-cause mortality, while cluster-2 had the lowest mortality rate. However, cluster-4 and − 5, which were characterized as five and four weight loss strategies, had a slightly attenuated effect. This may have something to do with the higher total energy they actually ate, despite they reported a weight loss strategy of lowering calories, eating less food or skipping meals. This is consistent with findings from another NHANES study, where participants reduced food consumption but no significant decreases were observed in calorie intake [12]. Moreover, among participants who used two weight-loss strategies, the greatest contributions to the lower risk of mortality were “ate less food” and “exercised”; among participants who used 3 to 4 strategies, the greatest contributions to the lower risk of mortality were “ate less food”, “exercised” and “drank a lot of water”; and among participants who used 5 or more strategies, the greatest contributions to reducing the risk of mortality were “ate less food”, “exercised”, “lowered calories” “drank a lot of water” and “ate less fat”. As more strategies are adopted, this trend of reduced risk of death is mainly related to the large proportion of “ate less food” and “exercised”, and the continuous increase of “lowered calories”, “ate less fat” and “drank a lot of water” (Figure S4). This may be related to their metabolic health benefits, especially when used in combination [37–39]. This is consistent with our conclusions in examining the mortality risk benefits of strategy combinations, that is, eating less food, exercising, drinking plenty of water, lowering calories and eating less fat is a better combination of strategies. For those with low long-term compliance, they can also choose two or more of them, especially cumulatively on the basis of “ate less food” and “exercised”. On this basis, limiting the actual intake of total energy is necessary.

At the same time, we should also pay attention to those factors that may affect the choice of strategies and their health effects, such as age, sex, physical condition and etc. A study by Kakinami et al. found that the use of strategies was related to income and age and that adults with higher household incomes were more likely to use recommended weight loss strategies (such as exercise or low fat) [40]. The 2018 U.S. Health Rankings Annual Report stated that obesity is highest among the U.S. adults aged 45 to 64, and they will benefit more from more aggressive weight loss strategies [41]. Ex-smokers who are prone to weight gain will also have a positive effect if they adopt more aggressive weight loss strategies [42]. These results also explain our finding that weight loss strategies are more strongly associated with a lower risk of all-cause mortality among participants aged 45–59 years, with better household income or past smoking.

### Strengths and limitations of study

The strengths of this study include the large and nationally representative sample obtained through strict inclusion and exclusion criteria, taking into account the complex survey design, including sample weights, sampling unit, and stratification. At the same time, we adjusted for a number of potential confounders, including socioeconomic status, lifestyle, and nutritional and health status. The novelty of this study is that we evaluated the association between using several weight loss strategies at the same time and mortality. By combining dietary adjustments, exercise routines, and pharmaceutical interventions, we have sought to uncover a more efficacious and safe approach, providing a fresh train of thought to improve the life expectancy of individuals with overweight and obesity.

However, our study also has several limitations. First, as an observational study, there is a chance of residual confounding, whereby unmeasured or unknown factors may influence the observed associations. Second, due to NHANES survey periods before 2005 failing to provide another 7 weight loss strategies, we cannot conduct a more comprehensive weight loss strategies and combination for analysis. We removed participants who adopted “other strategies” from the 14 weight loss strategies or included participants who adopted any of the newly added 7 weight loss strategies since 2005 as “other strategies” in sensitivity analyses, and the results did not change significantly. Third, NHANES did not collect data on the frequency or duration of weight loss attempts or strategies, and information on weight loss strategies and covariates was assessed only at a single time without measuring subsequent changes during follow-up. In addition, the weight loss strategies recalled by participants may have introduced misclassification bias, but this misclassification is likely nondifferential and presumably only biased our results toward the null. Fourth, there may be a reverse causal association in our study, so we excluded participants with cardiovascular disease and cancer at baseline or those who died within the first three years of follow-up or who did not report intentional weight loss but lost more than 10 pounds, and the results did not change substantially. Fifth, normal-weight participants following weight loss strategies may bring greater health effects. However, the results remained robust after we removed normal-weight participants. In addition, using BMI to define obesity may have certain limitations. When we analyzed obesity using waist circumference as the definition, the results remained the same. Finally, we cannot clearly distinguish that the weight change in this study was caused by intentional weight loss, but the willingness to take strategies to try to lose weight is certain.

## Conclusions

In conclusion, adopting two or more weight loss strategies was associated with a lower risk of all-cause mortality, even among those who gained weight. Eating less food, exercising, drinking plenty of water, lowering calories and eating less fat is a better combination of strategies. On this basis, limiting the actual intake of total energy is necessary. These findings are of high clinical and public health importance.

### Electronic supplementary material

Below is the link to the electronic supplementary material.

Supplementary Material 1

## Data Availability

Study protocol: Not available. Statistical code: Not available. Dataset: The datasets generated and/or analyzed during the current study are available from the NHANES website (https://www.cdc.gov/nchs/nhanes/index.htm).

## Abbreviations

NHANES: National Health and Nutrition Examination Survey
RCT: randomized clinical trial
