# Supplementary material for: Association of weight loss strategies with all-cause and specific-cause mortality: a prospective cohort study
Source: BMC Public Health. 2024 Aug 16;24:2234. doi: 10.1186/s12889-024-19472-z (PMC11330037; doi:10.1186/s12889-024-19472-z)
Supplement: Supplementary file 1 — Supplementary Material 1 [file 12889_2024_19472_MOESM1_ESM.docx]

**Contents**

[
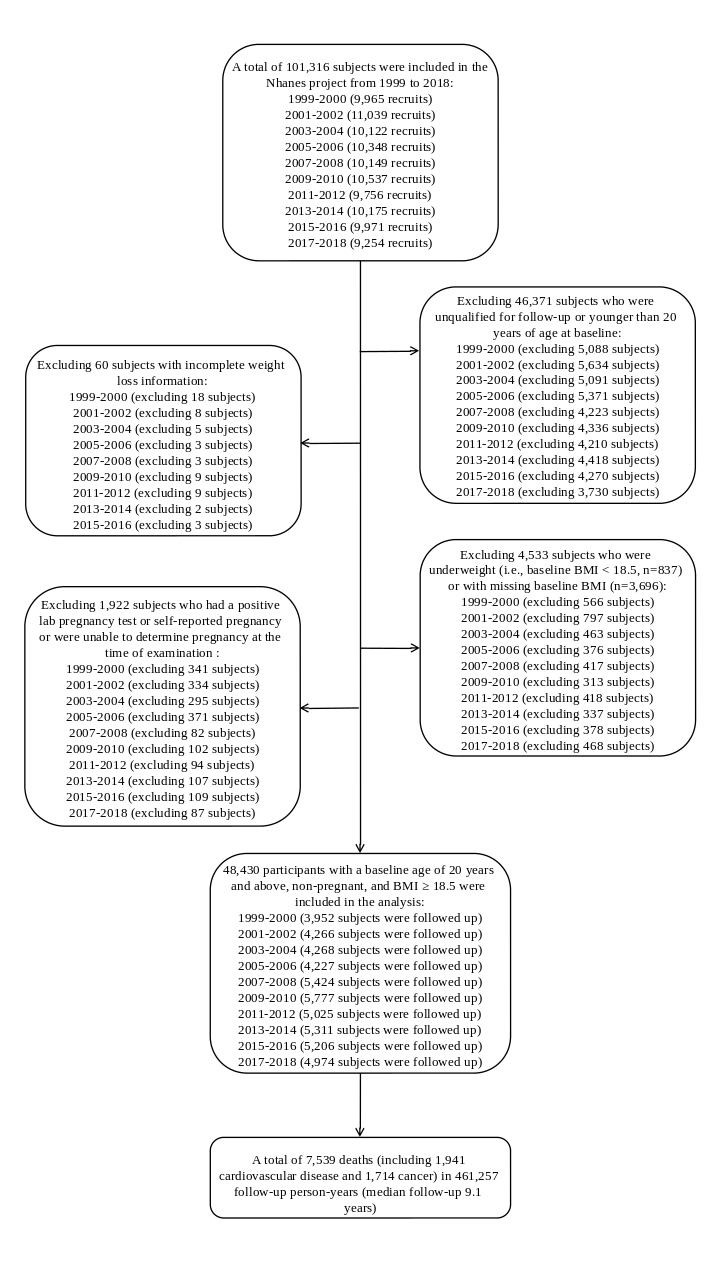

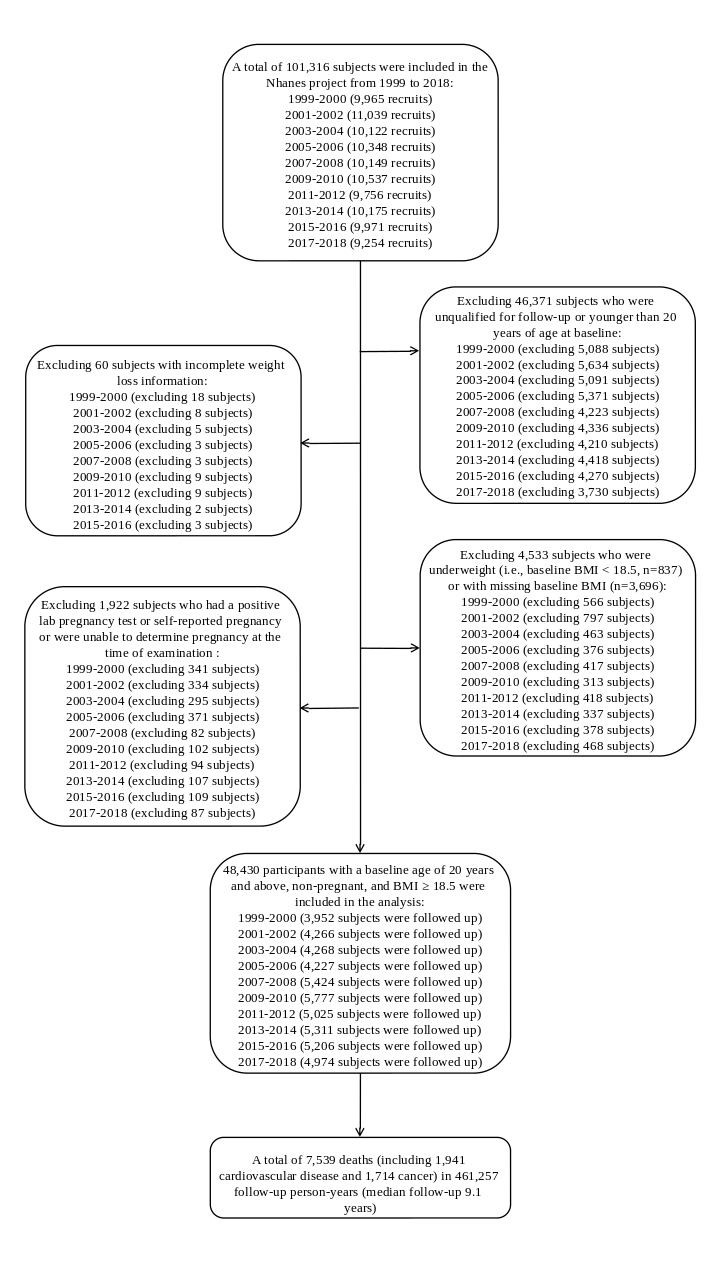

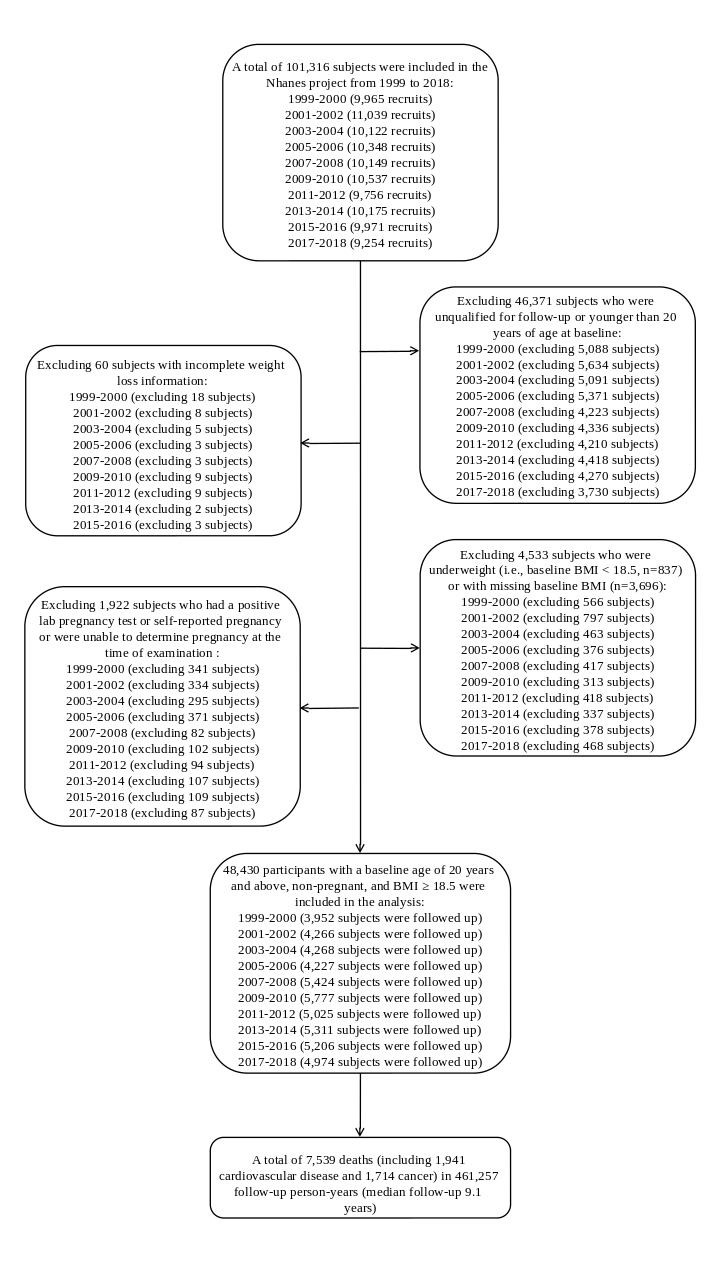
Figure S1. The inclusion screening process of the study participants 2](#_Toc170468651)

[Table S1. Number and percentage of participants with cumulative weight loss strategies and incidence of mortality 3](#_Toc170468652)

[Figure S2. Result of Latent Class Analysis 5](#_Toc170468653)

[Figure S3. Directed Acyclic Graph 6](#_Toc170468654)

[Table S2. Measurement of covariates in NHANES 8](#_Toc170468655)

[Table S3. The numbers (percentages) of participants with missing covariates 10](#_Toc170468656)

[Figure S4. Strategy distribution by the number of weight loss strategies 11](#_Toc170468657)

[Table S4. Latent classes and weight loss strategy distribution (N=15,449) 12](#_Toc170468658)

[Table S5. Hazard ratios (95% CIs) of mortality with the number of weight loss strategies in NHANES 1999-2018 (excluding participants with missing covariates, N=36,085) 13](#_Toc170468659)

[Table S6. Hazard ratios (95% CIs) of mortality with the number of weight loss strategies in NHANES 1999-2018 (excluding participants with coronary heart disease and cancer history, N=42,225) 14](#_Toc170468660)

[Table S7. Hazard ratios (95% CIs) of mortality with the number of weight loss strategies in NHANES 1999-2018 (excluding participants with mortality within 3 follow up years, N=46,805) 15](#_Toc170468661)

[Table S8. Hazard ratios (95% CIs) of mortality with the number of weight loss strategies in NHANES 1999-2018 (excluding participants with no intentional weight loss and weight loss more than 10 pounds since last year, N=44,964) 16](#_Toc170468662)

[Table S9. Hazard ratios (95% CIs) of mortality with the number of weight loss strategies in NHANES 1999-2018 (excluding participants with normal weight (BMI <25 kg/m^2^, N=34,580) 17](#_Toc170468663)

[Table S10. Hazard ratios (95% CIs) of mortality with the number of weight loss strategies in NHANES 1999-2018 (excluding participants with non-abdominal obesity [waist circumference: men <102cm, women <88cm], N=27,404) 18](#_Toc170468664)

[Table S11. Hazard ratios (95% CIs) of mortality with the number of weight loss strategies in NHANES 1999-2018 (excluding “other strategies” from the 14 weight loss strategies, N=48,430) 19](#_Toc170468665)

[Table S12. Hazard ratios (95% CIs) of mortality with the number of weight loss strategies in NHANES 1999-2018 (including additional adopted any of 7 weight loss strategies since 2005 as “other strategies”, N=48,430) 20](#_Toc170468666)

[Table S13. Hazard ratios (95% CIs) of mortality with the number of weight loss strategies in NHANES 1999-2018 (further adjusted survey rounds and country of birth, N=48,430) 21](#_Toc170468667)


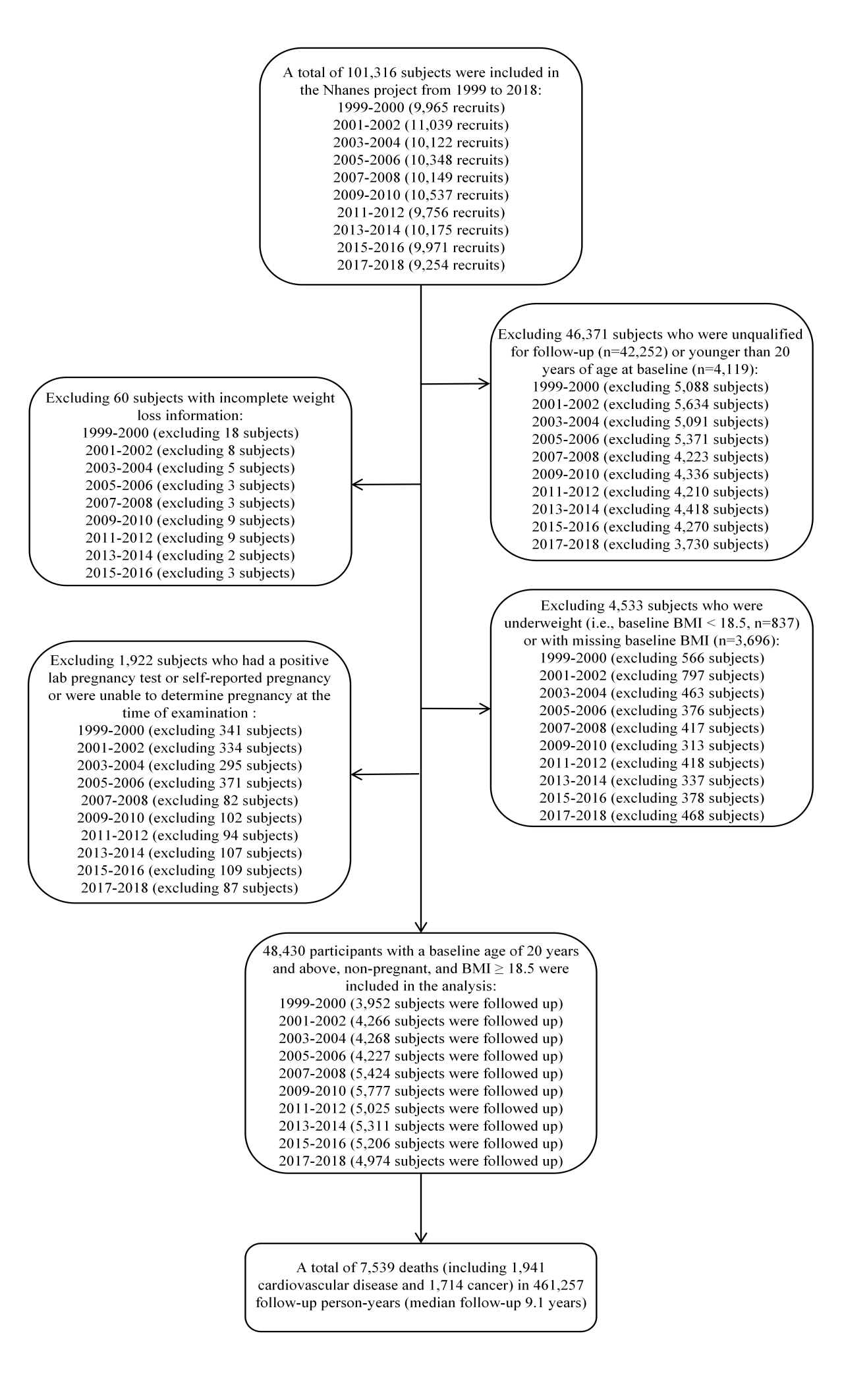

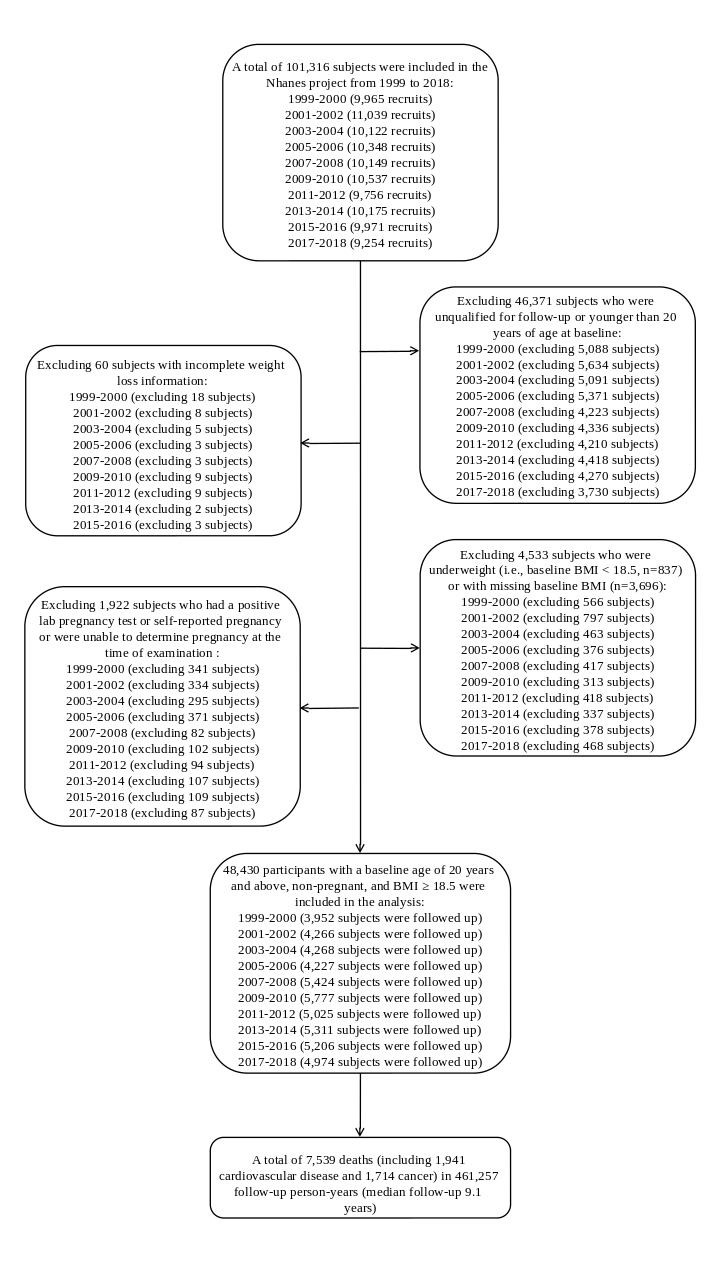

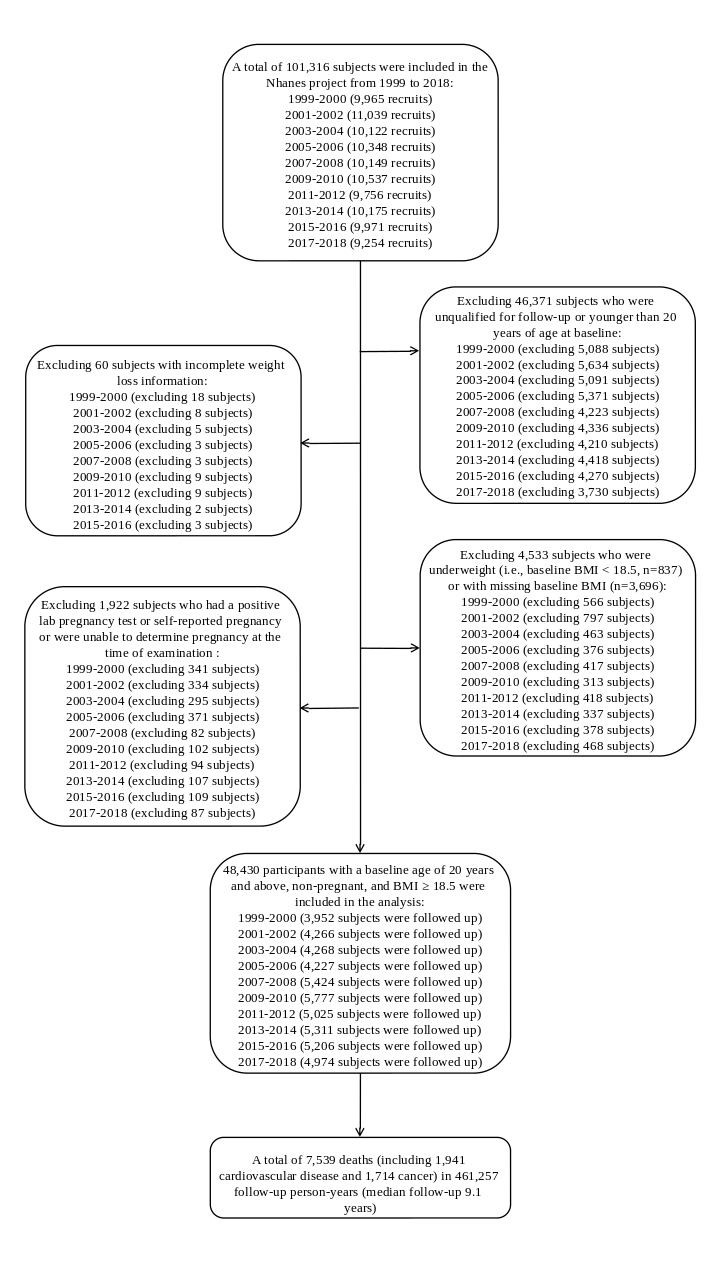

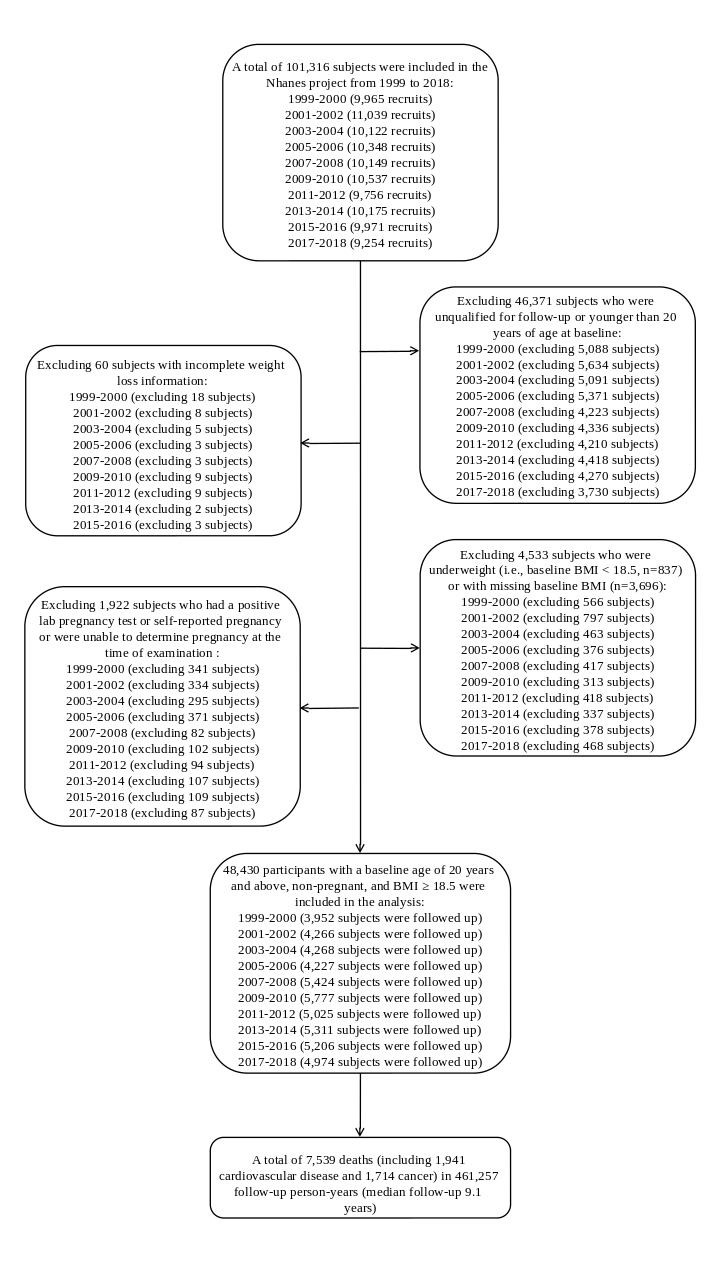
**Figure S1. The inclusion screening process of the study participants**

**Table S1. Number and percentage of participants with cumulative weight loss strategies and incidence of mortality**

| **Weight loss strategies** | **Number** | **Percentage (%)** | **Mortality rate (%)** |
| --- | --- | --- | --- |
| **The number of weight loss strategies** |  |  |  |
| 0 | 28365 | 58.57 | 19.10 |
| 1 | 4616 | 9.53 | 14.02 |
| 2 | 5043 | 10.41 | 11.42 |
| 3 | 4017 | 8.29 | 10.26 |
| 4 | 2864 | 5.91 | 10.16 |
| 5 | 1827 | 3.77 | 6.08 |
| 6 | 969 | 2.00 | 5.16 |
| 7 | 444 | 0.92 | 4.50 |
| 8 | 184 | 0.38 | 5.43 |
| 9 | 62 | 0.13 | 4.84 |
| 10 | 26 | 0.05 | 0.00 |
| 11 | 10 | 0.02 | 10.00 |
| 12 | 2 | 0.00 | 0.00 |
| 13 | 0 | 0.00 | 0.00 |
| 14 | 1 | 0.00 | 0.00 |
| **Cumulative weight loss strategies** |  |  |  |
| 0 | 28365 | 58.57 | 19.10 |
| 1 | 4616 | 9.53 | 14.02 |
| 2 | 5043 | 10.41 | 11.42 |
| 3-4 | 6881 | 14.21 | 10.22 |
| **≥**5 | 3525 | 7.28 | 5.53 |

| A  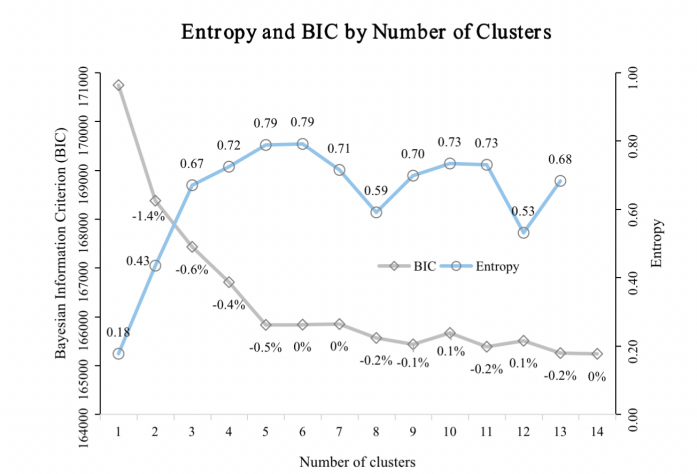 | B  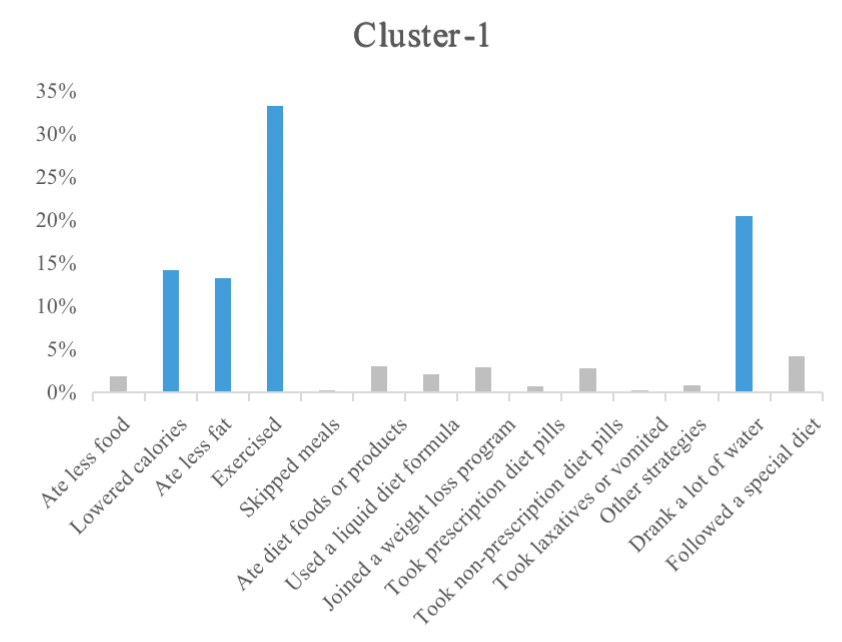 | C  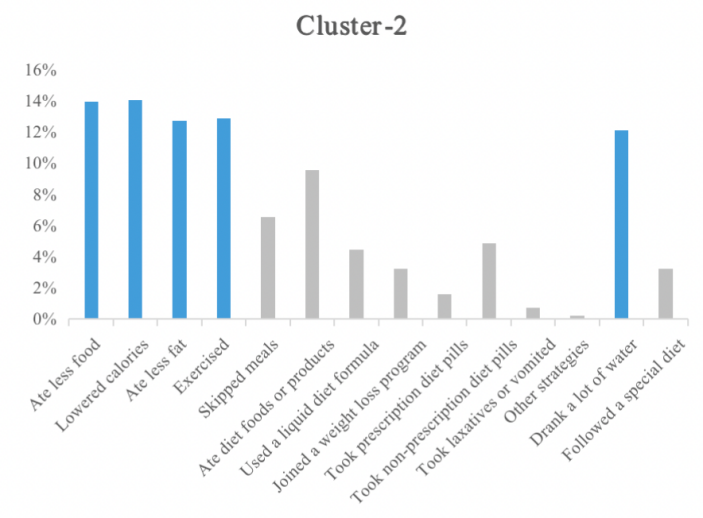 |
| --- | --- | --- |
| D  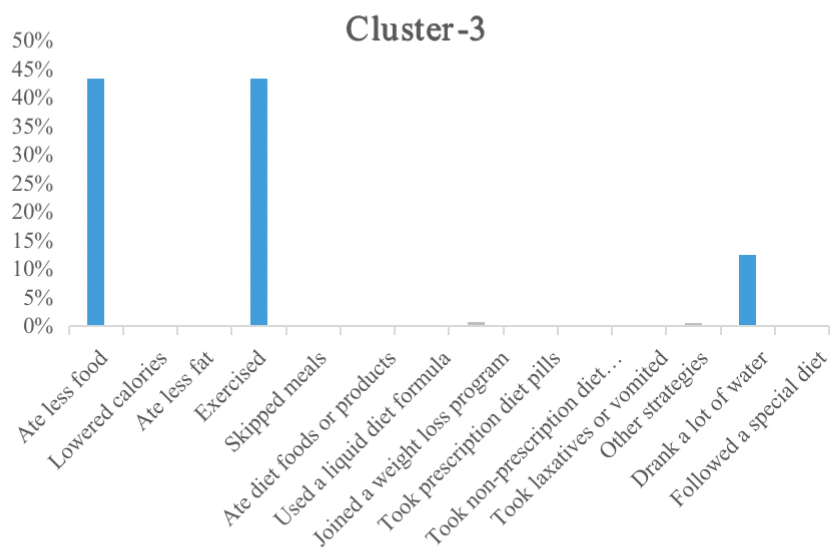 | E  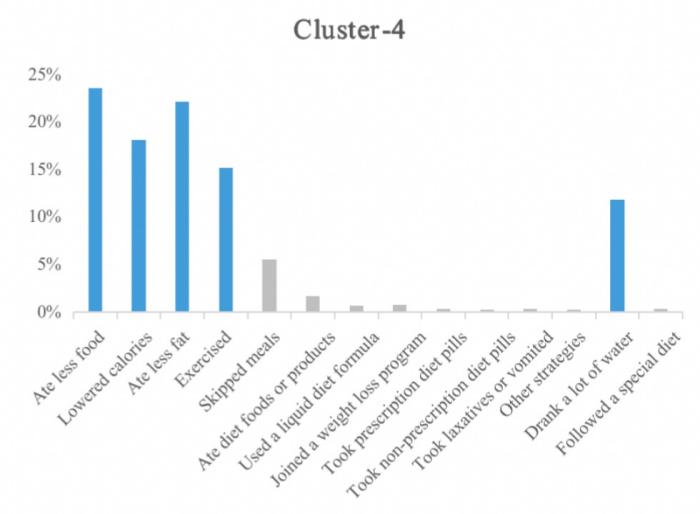 | F  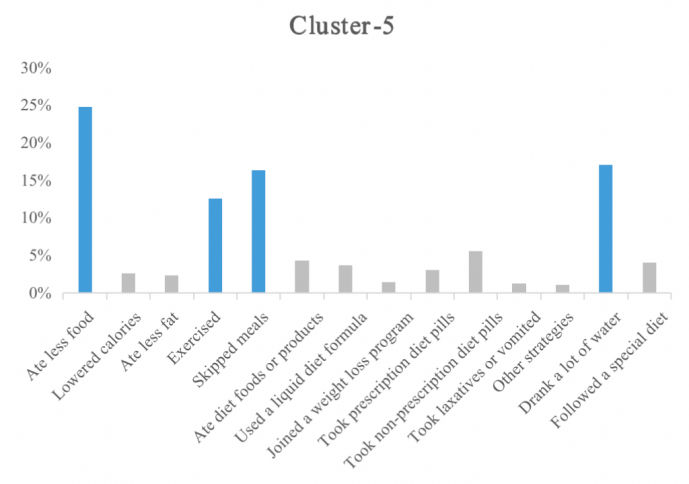 |

**Figure S2. Result of Latent Class Analysis**

(A): The trace plot of Bayesian information criterion (BIC) and entropy against the number of latent classes (or, clusters) by Latent Class Analysis (LCA). BIC and entropy were used for the model selection. BIC considered the log-likelihood of the class model and the number of model parameters. Lower BIC values indicated better model fit. (A) in the figure above shows that BIC continued to go down as more latent classes were added. However, the decrease leveled off after the five-latent-class solution. The entropy represented the accuracy of latent class, ranging from 0 to 1, and the closer to 1, the more accurate the classification. Entropy began with two clusters since it is not applicable to one-class models. As more latent classes were added, entropy increased, peaks after the five-latent-class solution, and then leveled off or decreased. Therefore, considering the improvement in statistical fit index (BIC), the class separation quality (entropy) and the interpretability of the clusters, the five-class solution was chosen as a concise and optimal representation of the weight loss strategy combinations of the study sample. (B), (C), (D), (E) and (F): Class membership distributions of the participants into the five latent classes for total samples. Percentages of weight loss strategies represent the proportion in each cluster. Blue bar means the percentages of weight loss strategies is greater than 10% in each cluster.


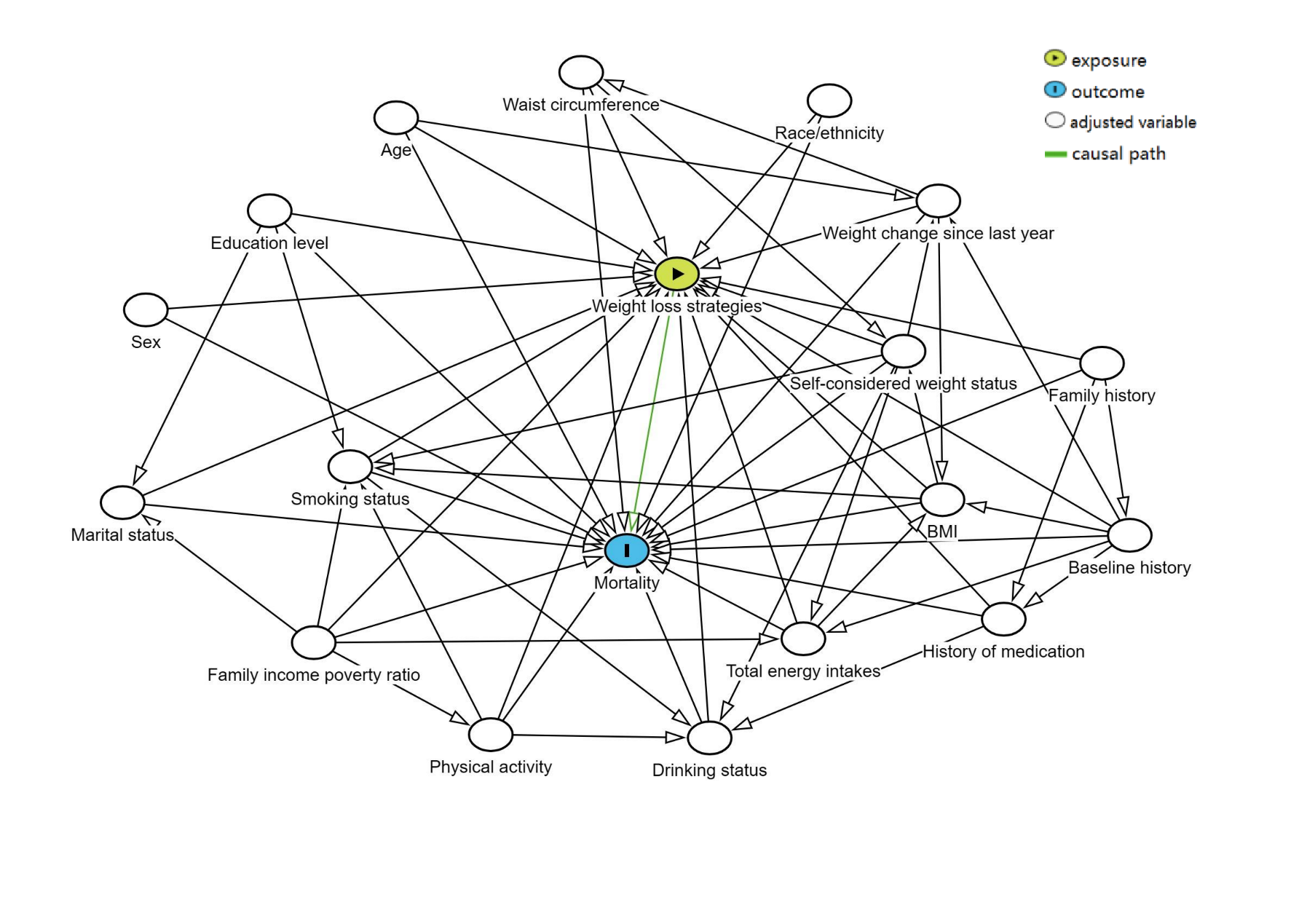


Figure S3. Directed Acyclic Graph

A directed acyclic graph represents associations between covariates and primary exposure and outcome. White circles represent ancestors of the exposure and outcome (ie, confounders), the blue circle represents the outcome, and the yellow circle represents the exposure. Green lines represent causal path. The minimally sufficient adjustment set represents covariates such that the adjustment for this set of variables will minimize confounding bias when estimating the association between the exposure and the outcome. The minimally sufficient adjustment set was determined using the DAGitty software*. The final minimally sufficient adjustment set comprised sex, age, race/ethnicity, education level, marital status, family income poverty ratio, weight change since last year, waist circumference, BMI, self-considered weight status, smoking status, drinking status, physical activity, total energy intakes, family history of prediabetes or diabetes and heart diseases, baseline history of diabetes, hypertension, high cholesterol, coronary heart disease and cancer, and history of medication for diabetes, hypertension and high cholesterol.

* Reference: Textor J, van der Zander B, Gilthorpe MS, et al. Robust causal inference using directed acyclic graphs: the R package 'dagitty'. *Int J Epidemiol* 2016;45(6):1887-94. doi: 10.1093/ije/dyw341

# Table S2. Measurement of covariates in NHANES

| **Covariates** | **Measurement** |
| --- | --- |
| Sex | Collected using the Sample Person and Family Demographics questionnaires |
| Age |  |
| Ethnicity |  |
| Education |  |
| Marital status |  |
| Family income poverty ratio |  |
| Weight change since last year (kg) | Collected from the Weight History section of the Sample Person Questionnaire, estimated as the difference value between current self-reported weight and self-reported weight one year ago |
| Waist circumference (cm) | Collected by trained health technicians in Mobile Examination Center |
| Body Mass Index **(**kg/m2) |  |
| Self-considered weight status | Collected from the Weight History section of the Sample Person Questionnaire, assessed by the question “How do you consider your weight?” |
| Smoking status | Those who did not have "Smoked at least 100 cigarettes in life" were none smokers. The question "Do you now smoke cigarettes?" was used to distinguish between current smokers and former smokers. |
| Drinking status | Considering the large missing data on alcohol intake and intake frequency in NHANES (1999-2018), drinking status (never, past or current) was measured through the question "In any one year, had at least 12 drinks of any type of alcoholic beverage?" and the question "Ever had a drink of any kind of alcohol?” |
| Physical activity | Taking into account the inconsistency and missing data of physical activity levels in NHANES (1999-2018), physical activity was measured by the question “In leisure time or at school over the past 30 days, did you do any moderate/vigorous activities for at least 10 minutes?” and the question “Do any moderate/vigorous-intensity sports, fitness, or recreational activities at least 10 minutes?” |
| Total energy intakes (kcal/d) | Assessed by one 24-hour dietary recall interview |
| Diabetes or prediabetes family history | Collected from the medical condition questionnaire with self-reported personal interview data |
| Heart disease family history |  |
| Diabetes history | Obtained by asking whether they had been informed of a disease by a doctor or health professional, assessed by the question “Ever told you have diabetes/ high blood pressure/ high cholesterol/ coronary heart disease/ cancer or malignancy?”* |
| Hypertension history |  |
| High cholesterol history |  |
| Coronary heart disease history |  |
| Cancer history |  |
| Diabetes prescribed medicine history | Collected by self-report questionnaire or verbal interviews |
| Hypertension prescribed medicine history |  |
| High cholesterol prescribed medicine history |  |

Variables of family income poverty ratio, weight change since last year, self-considered weight status, smoking status, drinking status, physical activity, total energy intakes, family history of prediabetes or diabetes and heart diseases, history of diabetes, hypertension, high cholesterol, coronary heart disease and cancer and history of medication for diabetes, hypertension and high cholesterol were self-reported.

* Reference: Chen C, Ye Y, Zhang Y, Pan XF, Pan A. Weight change across adulthood in relation to all cause and cause specific mortality: prospective cohort study. BMJ. 2019;367:l5584.http://doi.org/10.1136/bmj.l5584.

# Table S3. The numbers (percentages) of participants with missing covariates

| **Covariates** | **Count** | **Percentage (%)** |
| --- | --- | --- |
| Education | 60 | 0.12 |
| Marital status | 436 | 0.90 |
| Family income poverty ratio | 4306 | 8.89 |
| Weight change since last year (kg) | 1180 | 2.44 |
| Waist circumference (cm) | 1944 | 4.01 |
| Self-considered weight status | 136 | 0.28 |
| Alcohol consumption | 3950 | 8.16 |
| Total energy intakes (kcal) | 3025 | 6.25 |
| Diabetes or prediabetes family history | 965 | 1.99 |
| Heart disease family history | 1314 | 2.71 |
| Diabetes history | 31 | 0.06 |
| Hypertension history | 75 | 0.15 |
| High cholesterol history | 296 | 0.61 |
| Coronary heart disease history | 217 | 0.45 |
| Cancer history | 45 | 0.09 |
| Diabetes prescribed medicine history | 18 | 0.04 |
| Hypertension prescribed medicine history | 5 | 0.01 |
| High cholesterol prescribed medicine history | 13 | 0.03 |

Figure S4. Strategy distribution by the number of weight loss strategies

**Table S4. Latent classes and weight loss strategy distribution (N=15,449)**

| **Weight loss strategy** | **Cluster, n (%)** | | | | |
| --- | --- | --- | --- | --- | --- |
|  | **1** | **2** | **3** | **4** | **5** |
| Ate less food | 162 (1.9) | 1328 (13.9) | 2177 (43.4) | 5010 (23.5) | 2423 (24.8) |
| Lowered calories | 1196 (14.2) | 1338 (14.0) | 0 (0.0) | 3843 (18.0) | 252 (2.6) |
| Ate less fat | 1118 (13.2) | 1209 (12.7) | 0 (0.0) | 4718 (22.1) | 229 (2.3) |
| Exercised | 2800 (33.2) | 1224 (12.8) | 2177 (43.4) | 3224 (15.1) | 1231 (12.6) |
| Skipped meals | 23 (0.3) | 624 (6.5) | 0 (0.0) | 1178 (5.5) | 1600 (16.4) |
| Ate diet foods or products | 253 (3.0) | 908 (9.5) | 0 (0.0) | 352 (1.7) | 420 (4.3) |
| Used a liquid diet formula | 180 (2.1) | 426 (4.5) | 0 (0.0) | 142 (0.7) | 363 (3.7) |
| Joined a weight loss program | 247 (2.9) | 308 (3.2) | 33 (0.7) | 148 (0.7) | 137 (1.4) |
| Took prescription diet pills | 57 (0.7) | 150 (1.6) | 0 (0.0) | 23 (0.1) | 295 (3.0) |
| Took non-prescription diet pills | 239 (2.8) | 464 (4.9) | 0 (0.0) | 40 (0.2) | 543 (5.6) |
| Took laxatives or vomited | 26 (0.3) | 70 (0.7) | 0 (0.0) | 19 (0.1) | 120 (1.2) |
| Other strategies | 67 (0.8) | 22 (0.2) | 12 (0.2) | 52 (0.2) | 109 (1.1) |
| Drank a lot of water | 1724 (20.4) | 1151 (12.1) | 622 (12.4) | 2506 (11.8) | 1664 (17.0) |
| Followed a special diet | 353 (4.2) | 305 (3.2) | 0 (0.0) | 57 (0.3) | 397 (4.1) |
| **Mean total energy intakes (SD), kcal/d** | 2006.5 (886.4) | 2003.1 (917.1) | 2013.7 (895.4) | 2026.2 (939.3) | 2026.5 (969.7) |
| **Mean total carbohydrates intakes (SD), g/d** | 240.0 (128.5) | 238.2 (128.5) | 239.8 (128.5) | 243.7 (128.5) | 240.0 (128.5) |
| **Mean total fat intakes (SD), g/d** | 77.5 (46.8) | 78.0 (46.8) | 77.6 (46.8) | 77.5 (46.8) | 78.0 (46.8) |

# Table S5. Hazard ratios (95% CIs) of mortality with the number of weight loss strategies in NHANES 1999-2018 (excluding participants with missing covariates, N=36,085)

| **Mortality** | **The number of weight loss strategies** | | | | | ***P* value for trend** | **Per 1-strategy increment** |
| --- | --- | --- | --- | --- | --- | --- | --- |
|  | **0** | **1** | **2** | **3-4** | **≥5** |  |  |
| **All cause** |  |  |  |  |  |  |  |
| Incident rate‡ | 18.12 | 14.04 | 11.72 | 10.33 | 5.45 |  |  |
| Cases/N | 3671/20606 | 456/3408 | 424/3854 | 525/5375 | 149/2842 |  |  |
| Model1* | 1.00 (reference) | 0.90 (0.82 to 1.00) | 0.83 (0.75 to 0.91) | 0.81 (0.74 to 0.89) | 0.59 (0.50 to 0.70) | <0.00 | 0.91 (0.89 to 0.93) |
| Model2† | 1.00 (reference) | 0.97 (0.87 to 1.07) | 0.89 (0.80 to 0.99) | 0.90 (0.82 to 1.00) | 0.69 (0.58 to 0.82) | <0.00 | 0.95 (0.92 to 0.97) |
| **CVD** |  |  |  |  |  |  |  |
| Incident rate‡ | 4.69 | 4.31 | 3.01 | 2.93 | 0.99 |  |  |
| Cases/N | 949/20606 | 140/3408 | 109/3854 | 149/5375 | 27/2842 |  |  |
| Model1* | 1.00 (reference) | 1.11 (0.93 to 1.33) | 0.86 (0.71 to 1.05) | 0.96 (0.81 to 1.14) | 0.48 (0.32 to 0.70) | 0.01 | 0.94 (0.89 to 0.98) |
| Model2† | 1.00 (reference) | 1.15 (0.96 to 1.39) | 0.89 (0.72 to 1.09) | 1.02 (0.85 to 1.23) | 0.53 (0.36 to 0.79) | 0.08 | 0.95 (0.91 to 1.00) |
| **Cancer** |  |  |  |  |  |  |  |
| Incident rate‡ | 4.28 | 3.33 | 2.98 | 2.50 | 1.43 |  |  |
| Cases/N | 867/20606 | 108/3408 | 108/3854 | 127/5375 | 39/2842 |  |  |
| Model1* | 1.00 (reference) | 0.88 (0.72 to 1.07) | 0.86 (0.70 to 1.05) | 0.80 (0.66 to 0.96) | 0.60 (0.43 to 0.83) | 0.00 | 0.91 (0.87 to 0.96) |
| Model2† | 1.00 (reference) | 0.91 (0.74 to 1.12) | 0.89 (0.72 to 1.10) | 0.84 (0.69 to 1.02) | 0.65 (0.47 to 0.91) | 0.01 | 0.93 (0.88 to 0.98) |

* Model 1 was adjusted for baseline age (years, continuous) and sex (male or female).

† Model 2 was additionally adjusted by race/ethnicity (Hispanic, or non-Hispanic), education level (less than high school, high school or equivalent, or college or above), marital status (married, widowed, divorced, separated, or never married), family income poverty ratio (ratio, continuous), weight change since last year (pounds, continuous), waist circumference (cm, continuous), BMI (normal, overweight, or obesity), self-considered weight status (underweight, about the right weight, or overweight), smoking status (never, past, or current smoker), drinking status (never, past or current), physical activity (yes or no), total energy intakes (kcal/d, continuous), family history of prediabetes or diabetes and heart diseases (yes or no), baseline history of diabetes, hypertension, high cholesterol, coronary heart disease and cancer (yes or no), and history of medication for diabetes, hypertension and high cholesterol (yes or no).
‡ Incident rate per 1,000 person years.

# Table S6. Hazard ratios (95% CIs) of mortality with the number of weight loss strategies in NHANES 1999-2018 (excluding participants with coronary heart disease and cancer history, N=42,225)

| **Mortality** | **The number of weight loss strategies** | | | | | ***P* value for trend** | **Per 1-strategy increment** |
| --- | --- | --- | --- | --- | --- | --- | --- |
|  | **0** | **1** | **2** | **3-4** | **≥5** |  |  |
| **All cause** |  |  |  |  |  |  |  |
| Incident rate‡ | 15.19 | 12.05 | 9.36 | 8.74 | 4.61 |  |  |
| Cases/N | 3709/24514 | 469/4043 | 390/4406 | 503/6097 | 140/3165 |  |  |
| Model1* | 1.00 (reference) | 0.91 (0.82 to 1.00) | 0.82 (0.74 to 0.91) | 0.82 (0.75 to 0.91) | 0.60 (0.51 to 0.72) | <0.00 | 0.91 (0.89 to 0.94) |
| Model2† | 1.00 (reference) | 0.95 (0.86 to 1.05) | 0.90 (0.81 to 1.01) | 0.90 (0.82 to 1.00) | 0.71 (0.60 to 0.85) | <0.00 | 0.95 (0.92 to 0.97) |
| **CVD** |  |  |  |  |  |  |  |
| Incident rate‡ | 3.89 | 3.16 | 2.50 | 2.41 | 0.89 |  |  |
| Cases/N | 951/24514 | 123/4043 | 104/4406 | 139/6097 | 27/3165 |  |  |
| Model1* | 1.00 (reference) | 0.95 (0.79 to 1.15) | 0.90 (0.74 to 1.11) | 0.95 (0.79 to 1.14) | 0.51 (0.35 to 0.75) | 0.00 | 0.94 (0.89 to 0.98) |
| Model2† | 1.00 (reference) | 0.96 (0.79 to 1.17) | 0.97 (0.78 to 1.19) | 1.01 (0.83 to 1.23) | 0.59 (0.39 to 0.87) | 0.17 | 0.96 (0.91 to 1.02) |
| **Cancer** |  |  |  |  |  |  |  |
| Incident rate‡ | 3.06 | 2.70 | 1.99 | 1.88 | 1.02 |  |  |
| Cases/N | 748/24514 | 105/4043 | 83/4406 | 108/6097 | 31/3165 |  |  |
| Model1* | 1.00 (reference) | 0.99 (0.80 to 1.21) | 0.83 (0.66 to 1.05) | 0.84 (0.69 to 1.03) | 0.61 (0.43 to 0.88) | 0.00 | 0.92 (0.88 to 0.97) |
| Model2† | 1.00 (reference) | 1.00 (0.81 to 1.23) | 0.86 (0.68 to 1.09) | 0.88 (0.71 to 1.09) | 0.68 (0.47 to 0.98) | 0.03 | 0.94 (0.88 to 0.99) |

* Model 1 was adjusted for baseline age (years, continuous) and sex (male or female).

† Model 2 was additionally adjusted by race/ethnicity (Hispanic, or non-Hispanic), education level (less than high school, high school or equivalent, or college or above), marital status (married, widowed, divorced, separated, or never married), family income poverty ratio (ratio, continuous), weight change since last year (pounds, continuous), waist circumference (cm, continuous), BMI (normal, overweight, or obesity), self-considered weight status (underweight, about the right weight, or overweight), smoking status (never, past, or current smoker), drinking status (never, past or current), physical activity (yes or no), total energy intakes (kcal/d, continuous), family history of prediabetes or diabetes and heart diseases (yes or no), baseline history of diabetes, hypertension, high cholesterol, coronary heart disease and cancer (yes or no), and history of medication for diabetes, hypertension and high cholesterol (yes or no).
‡ Incident rate per 1,000 person years.

# Table S7. Hazard ratios (95% CIs) of mortality with the number of weight loss strategies in NHANES 1999-2018 (excluding participants with mortality within 3 follow up years, N=46,805)

| **Mortality** | **The number of weight loss strategies** | | | | | ***P* value for trend** | **Per 1-strategy increment** |
| --- | --- | --- | --- | --- | --- | --- | --- |
|  | **0** | **1** | **2** | **3-4** | **≥5** |  |  |
| **All cause** |  |  |  |  |  |  |  |
| Incident rate‡ | 15.49 | 11.82 | 10.13 | 9.03 | 4.56 |  |  |
| Cases/N | 4201/27148 | 510/4479 | 472/4939 | 578/6756 | 153/3483 |  |  |
| Model1* | 1.00 (reference) | 0.90 (0.82 to 0.99) | 0.85 (0.77 to 0.94) | 0.83 (0.76 to 0.91) | 0.59 (0.51 to 0.70) | <0.00 | 0.92 (0.90 to 0.94) |
| Model2† | 1.00 (reference) | 0.93 (0.85 to 1.02) | 0.91 (0.82 to 1.00) | 0.90 (0.82 to 0.99) | 0.68 (0.58 to 0.81) | <0.00 | 0.95 (0.92 to 0.97) |
| **CVD** |  |  |  |  |  |  |  |
| Incident rate‡ | 3.97 | 3.27 | 2.64 | 2.48 | 0.83 |  |  |
| Cases/N | 1077/27148 | 141/4479 | 123/4939 | 159/6756 | 28/3483 |  |  |
| Model1* | 1.00 (reference) | 1.00 (0.84 to 1.19) | 0.90 (0.75 to 1.09) | 0.95 (0.81 to 1.13) | 0.48 (0.33 to 0.70) | 0.01 | 0.94 (0.90 to 0.98) |
| Model2† | 1.00 (reference) | 0.97 (0.81 to 1.17) | 0.90 (0.74 to 1.10) | 0.96 (0.80 to 1.15) | 0.51 (0.35 to 0.75) | 0.02 | 0.94 (0.90 to 0.99) |
| **Cancer** |  |  |  |  |  |  |  |
| Incident rate‡ | 3.25 | 2.66 | 2.43 | 2.14 | 0.98 |  |  |
| Cases/N | 881/27148 | 115/4479 | 113/4939 | 137/6756 | 33/3483 |  |  |
| Model1* | 1.00 (reference) | 0.94 (0.77 to 1.14) | 0.93 (0.76 to 1.13) | 0.89 (0.74 to 1.07) | 0.55 (0.38 to 0.77) | 0.00 | 0.93 (0.89 to 0.98) |
| Model2† | 1.00 (reference) | 0.93 (0.76 to 1.13) | 0.94 (0.76 to 1.15) | 0.91 (0.75 to 1.10) | 0.58 (0.40 to 0.83) | 0.02 | 0.94 (0.89 to 0.99) |

* Model 1 was adjusted for baseline age (years, continuous) and sex (male or female).

† Model 2 was additionally adjusted by race/ethnicity (Hispanic, or non-Hispanic), education level (less than high school, high school or equivalent, or college or above), marital status (married, widowed, divorced, separated, or never married), family income poverty ratio (ratio, continuous), weight change since last year (pounds, continuous), waist circumference (cm, continuous), BMI (normal, overweight, or obesity), self-considered weight status (underweight, about the right weight, or overweight), smoking status (never, past, or current smoker), drinking status (never, past or current), physical activity (yes or no), total energy intakes (kcal/d, continuous), family history of prediabetes or diabetes and heart diseases (yes or no), baseline history of diabetes, hypertension, high cholesterol, coronary heart disease and cancer (yes or no), and history of medication for diabetes, hypertension and high cholesterol (yes or no).
‡ Incident rate per 1,000 person year.

# Table S8. Hazard ratios (95% CIs) of mortality with the number of weight loss strategies in NHANES 1999-2018 (excluding participants with no intentional weight loss and weight loss more than 10 pounds since last year, N=44,964)

| **Mortality** | **The number of weight loss strategies** | | | | | ***P* value for trend** | **Per 1-strategy increment** |
| --- | --- | --- | --- | --- | --- | --- | --- |
|  | **0** | **1** | **2** | **3-4** | **≥5** |  |  |
| **All cause** |  |  |  |  |  |  |  |
| Incident rate‡ | 18.58 | 14.63 | 12.16 | 10.79 | 5.63 |  |  |
| Cases/N | 4628/25456 | 616/4472 | 551/4889 | 674/6688 | 186/3459 |  |  |
| Model1* | 1.00 (reference) | 0.93 (0.85 to 1.01) | 0.85 (0.78 to 0.93) | 0.84 (0.78 to 0.92) | 0.63 (0.54 to 0.73) | <0.00 | 0.92 (0.90 to 0.94) |
| Model2† | 1.00 (reference) | 0.96 (0.88 to 1.05) | 0.92 (0.84 to 1.01) | 0.92 (0.84 to 1.00) | 0.73 (0.63 to 0.85) | <0.00 | 0.95 (0.93 to 0.98) |
| **CVD** |  |  |  |  |  |  |  |
| Incident rate‡ | 4.70 | 4.04 | 3.22 | 3.07 | 1.00 |  |  |
| Cases/N | 1171/25456 | 170/4472 | 146/4889 | 192/6688 | 33/3459 |  |  |
| Model1* | 1.00 (reference) | 1.05 (0.89 to 1.23) | 0.94 (0.79 to 1.11) | 1.02 (0.88 to 1.19) | 0.50 (0.35 to 0.71) | 0.03 | 0.96 (0.92 to 1.00) |
| Model2† | 1.00 (reference) | 1.04 (0.88 to 1.23) | 0.97 (0.81 to 1.16) | 1.06 (0.90 to 1.25) | 0.56 (0.39 to 0.80) | 0.21 | 0.97 (0.93 to 1.02) |
| **Cancer** |  |  |  |  |  |  |  |
| Incident rate‡ | 4.21 | 3.42 | 3.00 | 2.51 | 1.36 |  |  |
| Cases/N | 1048/25456 | 144/4472 | 136/4889 | 157/6688 | 45/3459 |  |  |
| Model1* | 1.00 (reference) | 0.93 (0.78 to 1.11) | 0.89 (0.75 to 1.07) | 0.83 (0.70 to 0.98) | 0.61 (0.45 to 0.82) | <0.00 | 0.92 (0.88 to 0.96) |
| Model2† | 1.00 (reference) | 0.93 (0.78 to 1.12) | 0.91 (0.76 to 1.10) | 0.85 (0.71 to 1.02) | 0.65 (0.48 to 0.88) | 0.00 | 0.93 (0.89 to 0.98) |

* Model 1 was adjusted for baseline age (years, continuous) and sex (male or female).

† Model 2 was additionally adjusted by race/ethnicity (Hispanic, or non-Hispanic), education level (less than high school, high school or equivalent, or college or above), marital status (married, widowed, divorced, separated, or never married), family income poverty ratio (ratio, continuous), weight change since last year (pounds, continuous), waist circumference (cm, continuous), BMI (normal, overweight, or obesity), self-considered weight status (underweight, about the right weight, or overweight), smoking status (never, past, or current smoker), drinking status (never, past or current), physical activity (yes or no), total energy intakes (kcal/d, continuous), family history of prediabetes or diabetes and heart diseases (yes or no), baseline history of diabetes, hypertension, high cholesterol, coronary heart disease and cancer (yes or no), and history of medication for diabetes, hypertension and high cholesterol (yes or no).
‡ Incident rate per 1,000 person years.

# Table S9. Hazard ratios (95% CIs) of mortality with the number of weight loss strategies in NHANES 1999-2018 (excluding participants with normal weight (BMI <25 kg/m^2^, N=34,580)

| **Mortality** | **The number of weight loss strategies** | | | | | ***P* value for trend** | **Per 1-strategy increment** |
| --- | --- | --- | --- | --- | --- | --- | --- |
|  | **0** | **1** | **2** | **3-4** | **≥5** |  |  |
| **All cause** |  |  |  |  |  |  |  |
| Incident rate‡ | 20.23 | 15.94 | 13.16 | 11.69 | 5.97 |  |  |
| Cases/N | 3355/17227 | 585/3938 | 524/4342 | 644/5980 | 174/3093 |  |  |
| Model1* | 1.00 (reference) | 0.93 (0.85 to 1.02) | 0.87 (0.79 to 0.95) | 0.84 (0.77 to 0.92) | 0.62 (0.53 to 0.72) | <0.00 | 0.92 (0.90 to 0.94) |
| Model2† | 1.00 (reference) | 0.95 (0.86 to 1.03) | 0.91 (0.82 to 0.99) | 0.89 (0.82 to 0.97) | 0.69 (0.59 to 0.81) | <0.00 | 0.95 (0.92 to 0.97) |
| **CVD** |  |  |  |  |  |  |  |
| Incident rate‡ | 5.42 | 4.39 | 3.64 | 3.39 | 1.10 |  |  |
| Cases/N | 899/17227 | 161/3938 | 145/4342 | 187/5980 | 32/3093 |  |  |
| Model1* | 1.00 (reference) | 0.98 (0.83 to 1.16) | 0.93 (0.78 to 1.11) | 0.97 (0.83 to 1.13) | 0.47 (0.33 to 0.67) | 0.01 | 0.94 (0.90 to 0.98) |
| Model2† | 1.00 (reference) | 0.99 (0.83 to 1.17) | 0.96 (0.80 to 1.15) | 1.00 (0.85 to 1.19) | 0.51 (0.36 to 0.74) | 0.05 | 0.96 (0.91 to 1.00) |
| **Cancer** |  |  |  |  |  |  |  |
| Incident rate‡ | 4.71 | 3.70 | 3.11 | 2.76 | 1.54 |  |  |
| Cases/N | 781/17227 | 136/3938 | 124/4342 | 152/5980 | 45/3093 |  |  |
| Model1* | 1.00 (reference) | 0.91 (0.76 to 1.10) | 0.85 (0.70 to 1.03) | 0.82 (0.69 to 0.98) | 0.63 (0.46 to 0.85) | <0.00 | 0.92 (0.88 to 0.96) |
| Model2† | 1.00 (reference) | 0.91 (0.75 to 1.09) | 0.86 (0.71 to 1.05) | 0.86 (0.72 to 1.03) | 0.68 (0.50 to 0.93) | 0.01 | 0.93 (0.89 to 0.98) |

* Model 1 was adjusted for baseline age (years, continuous) and sex (male or female).

† Model 2 was additionally adjusted by race/ethnicity (Hispanic, or non-Hispanic), education level (less than high school, high school or equivalent, or college or above), marital status (married, widowed, divorced, separated, or never married), family income poverty ratio (ratio, continuous), weight change since last year (pounds, continuous), waist circumference (cm, continuous), BMI (normal, overweight, or obesity), self-considered weight status (underweight, about the right weight, or overweight), smoking status (never, past, or current smoker), drinking status (never, past or current), physical activity (yes or no), total energy intakes (kcal/d, continuous), family history of prediabetes or diabetes and heart diseases (yes or no), baseline history of diabetes, hypertension, high cholesterol, coronary heart disease and cancer (yes or no), and history of medication for diabetes, hypertension and high cholesterol (yes or no).
‡ Incident rate per 1,000 person years.

# Table S10. Hazard ratios (95% CIs) of mortality with the number of weight loss strategies in NHANES 1999-2018 (excluding participants with non-abdominal obesity [waist circumference: men <102cm, women <88cm], N=27,404)

| **Mortality** | **The number of weight loss strategies** | | | | | ***P* value for trend** | **Per 1-strategy increment** |
| --- | --- | --- | --- | --- | --- | --- | --- |
|  | **0** | **1** | **2** | **3-4** | **≥5** |  |  |
| **All cause** |  |  |  |  |  |  |  |
| Incident rate‡ | 24.81 | 18.04 | 14.89 | 12.90 | 6.96 |  |  |
| Cases/N | 2955/12819 | 542/3290 | 486/3629 | 583/4994 | 173/2672 |  |  |
| Model1* | 1.00 (reference) | 0.92 (0.84 to 1.01) | 0.83 (0.76 to 0.92) | 0.80 (0.73 to 0.87) | 0.62 (0.53 to 0.72) | <0.00 | 0.91 (0.89 to 0.93) |
| Model2† | 1.00 (reference) | 0.95 (0.86 to 1.04) | 0.89 (0.81 to 0.98) | 0.86 (0.78 to 0.94) | 0.73 (0.62 to 0.85) | <0.00 | 0.94 (0.92 to 0.96) |
| **CVD** |  |  |  |  |  |  |  |
| Incident rate‡ | 6.66 | 4.69 | 4.04 | 3.72 | 1.37 |  |  |
| Cases/N | 793/12819 | 141/3290 | 132/3629 | 168/4994 | 34/2672 |  |  |
| Model1* | 1.00 (reference) | 0.92 (0.77 to 1.10) | 0.88 (0.73 to 1.06) | 0.91 (0.77 to 1.08) | 0.51 (0.36 to 0.72) | 0.00 | 0.93 (0.89 to 0.97) |
| Model2† | 1.00 (reference) | 0.92 (0.76 to 1.10) | 0.89 (0.74 to 1.08) | 0.93 (0.78 to 1.11) | 0.56 (0.39 to 0.80) | 0.01 | 0.94 (0.89 to 0.99) |
| **Cancer** |  |  |  |  |  |  |  |
| Incident rate‡ | 5.45 | 4.23 | 3.74 | 3.01 | 1.69 |  |  |
| Cases/N | 649/12819 | 127/3290 | 122/3629 | 136/4994 | 42/2672 |  |  |
| Model1* | 1.00 (reference) | 0.94 (0.77 to 1.13) | 0.89 (0.73 to 1.08) | 0.79 (0.66 to 0.96) | 0.60 (0.44 to 0.83) | <0.00 | 0.91 (0.87 to 0.96) |
| Model2† | 1.00 (reference) | 0.95 (0.78 to 1.16) | 0.94 (0.77 to 1.15) | 0.84 (0.69 to 1.02) | 0.68 (0.49 to 0.94) | 0.01 | 0.94 (0.89 to 0.99) |

* Model 1 was adjusted for baseline age (years, continuous) and sex (male or female).

† Model 2 was additionally adjusted by race/ethnicity (Hispanic, or non-Hispanic), education level (less than high school, high school or equivalent, or college or above), marital status (married, widowed, divorced, separated, or never married), family income poverty ratio (ratio, continuous), weight change since last year (pounds, continuous), waist circumference (cm, continuous), BMI (normal, overweight, or obesity), self-considered weight status (underweight, about the right weight, or overweight), smoking status (never, past, or current smoker), drinking status (never, past or current), physical activity (yes or no), total energy intakes (kcal/d, continuous), family history of prediabetes or diabetes and heart diseases (yes or no), baseline history of diabetes, hypertension, high cholesterol, coronary heart disease and cancer (yes or no), and history of medication for diabetes, hypertension and high cholesterol (yes or no).
‡ Incident rate per 1,000 person years.

# Table S11. Hazard ratios (95% CIs) of mortality with the number of weight loss strategies in NHANES 1999-2018 (excluding “other strategies” from the 14 weight loss strategies, N=48,430)

| **Mortality** | **The number of weight loss strategies** | | | | | ***P* value for trend** | **Per 1-strategy increment** |
| --- | --- | --- | --- | --- | --- | --- | --- |
|  | **0** | **1** | **2** | **3-4** | **≥5** |  |  |
| **All cause** |  |  |  |  |  |  |  |
| Incident rate‡ | 19.85 | 14.61 | 12.33 | 10.93 | 5.72 |  |  |
| Cases/N | 5445/28468 | 630/4586 | 575/5032 | 699/6862 | 190/3482 |  |  |
| Model1* | 1.00 (reference) | 0.88 (0.81 to 0.95) | 0.81 (0.74 to 0.88) | 0.81 (0.74 to 0.87) | 0.59 (0.51 to 0.69) | <0.00 | 0.91 (0.89 to 0.93) |
| Model2† | 1.00 (reference) | 0.92 (0.84 to 1.00) | 0.88 (0.81 to 0.97) | 0.89 (0.82 to 0.97) | 0.70 (0.60 to 0.81) | <0.00 | 0.94 (0.92 to 0.96) |
| **CVD** |  |  |  |  |  |  |  |
| Incident rate‡ | 5.05 | 3.92 | 3.35 | 3.06 | 1.05 |  |  |
| Cases/N | 1385/28468 | 169/4586 | 156/5032 | 196/6862 | 35/3482 |  |  |
| Model1* | 1.00 (reference) | 0.95 (0.81 to 1.12) | 0.90 (0.76 to 1.07) | 0.95 (0.82 to 1.11) | 0.49 (0.35 to 0.68) | 0.00 | 0.94 (0.90 to 0.98) |
| Model2† | 1.00 (reference) | 0.95 (0.80 to 1.12) | 0.93 (0.78 to 1.11) | 0.99 (0.84 to 1.16) | 0.53 (0.38 to 0.75) | 0.03 | 0.95 (0.91 to 1.00) |
| **Cancer** |  |  |  |  |  |  |  |
| Incident rate‡ | 4.43 | 3.43 | 3.00 | 2.58 | 1.41 |  |  |
| Cases/N | 1214/28468 | 148/4586 | 140/5032 | 165/6862 | 47/3482 |  |  |
| Model1* | 1.00 (reference) | 0.90 (0.76 to 1.07) | 0.85 (0.72 to 1.02) | 0.82 (0.70 to 0.97) | 0.61 (0.45 to 0.81) | <0.00 | 0.92 (0.88 to 0.96) |
| Model2† | 1.00 (reference) | 0.91 (0.76 to 1.09) | 0.89 (0.74 to 1.07) | 0.86 (0.72 to 1.03) | 0.66 (0.49 to 0.89) | 0.00 | 0.93 (0.89 to 0.98) |

* Model 1 was adjusted for baseline age (years, continuous) and sex (male or female).

† Model 2 was additionally adjusted by race/ethnicity (Hispanic, or non-Hispanic), education level (less than high school, high school or equivalent, or college or above), marital status (married, widowed, divorced, separated, or never married), family income poverty ratio (ratio, continuous), weight change since last year (pounds, continuous), waist circumference (cm, continuous), BMI (normal, overweight, or obesity), self-considered weight status (underweight, about the right weight, or overweight), smoking status (never, past, or current smoker), drinking status (never, past or current), physical activity (yes or no), total energy intakes (kcal/d, continuous), family history of prediabetes or diabetes and heart diseases (yes or no), baseline history of diabetes, hypertension, high cholesterol, coronary heart disease and cancer (yes or no), and history of medication for diabetes, hypertension and high cholesterol (yes or no).
‡ Incident rate per 1,000 person years.

# Table S12. Hazard ratios (95% CIs) of mortality with the number of weight loss strategies in NHANES 1999-2018 (including additional adopted any of 7 weight loss strategies since 2005 as “other strategies”, N=48,430)

| **Mortality** | **The number of weight loss strategies** | | | | | ***P* value for trend** | **Per 1-strategy increment** |
| --- | --- | --- | --- | --- | --- | --- | --- |
|  | **0** | **1** | **2** | **3-4** | **≥5** |  |  |
| **All cause** |  |  |  |  |  |  |  |
| Incident rate‡ | 19.88 | 15.58 | 13.44 | 10.88 | 6.38 |  |  |
| Cases/N | 5384/28004 | 574/3615 | 576/4381 | 721/7215 | 284/5215 |  |  |
| Model1* | 1.00 (reference) | 0.90 (0.83 to 0.98) | 0.84 (0.77 to 0.92) | 0.79 (0.73 to 0.85) | 0.65 (0.58 to 0.73) | <0.00 | 0.91 (0.90 to 0.93) |
| Model2† | 1.00 (reference) | 0.94 (0.86 to 1.03) | 0.93 (0.85 to 1.02) | 0.85 (0.78 to 0.92) | 0.76 (0.67 to 0.86) | <0.00 | 0.94 (0.92 to 0.96) |
| **CVD** |  |  |  |  |  |  |  |
| Incident rate‡ | 5.04 | 4.37 | 3.50 | 3.18 | 1.24 |  |  |
| Cases/N | 1364/28004 | 161/3615 | 150/4381 | 211/7215 | 55/5215 |  |  |
| Model1* | 1.00 (reference) | 1.03 (0.87 to 1.21) | 0.90 (0.76 to 1.07) | 0.97 (0.84 to 1.12) | 0.56 (0.43 to 0.73) | 0.00 | 0.94 (0.91 to 0.98) |
| Model2† | 1.00 (reference) | 1.02 (0.86 to 1.21) | 0.95 (0.80 to 1.13) | 0.99 (0.84 to 1.15) | 0.60 (0.45 to 0.79) | 0.02 | 0.95 (0.91 to 0.99) |
| **Cancer** |  |  |  |  |  |  |  |
| Incident rate‡ | 4.44 | 3.39 | 3.27 | 2.66 | 1.57 |  |  |
| Cases/N | 1203/28004 | 125/3615 | 140/4381 | 176/7215 | 70/5215 |  |  |
| Model1* | 1.00 (reference) | 0.87 (0.72 to 1.04) | 0.89 (0.75 to 1.06) | 0.82 (0.70 to 0.96) | 0.66 (0.51 to 0.84) | <0.00 | 0.92 (0.89 to 0.96) |
| Model2† | 1.00 (reference) | 0.87 (0.72 to 1.05) | 0.93 (0.78 to 1.12) | 0.85 (0.71 to 1.00) | 0.72 (0.56 to 0.93) | 0.01 | 0.94 (0.90 to 0.98) |

* Model 1 was adjusted for baseline age (years, continuous) and sex (male or female).

† Model 2 was additionally adjusted by race/ethnicity (Hispanic, or non-Hispanic), education level (less than high school, high school or equivalent, or college or above), marital status (married, widowed, divorced, separated, or never married), family income poverty ratio (ratio, continuous), weight change since last year (pounds, continuous), waist circumference (cm, continuous), BMI (normal, overweight, or obesity), self-considered weight status (underweight, about the right weight, or overweight), smoking status (never, past, or current smoker), drinking status (never, past or current), physical activity (yes or no), total energy intakes (kcal/d, continuous), family history of prediabetes or diabetes and heart diseases (yes or no), baseline history of diabetes, hypertension, high cholesterol, coronary heart disease and cancer (yes or no), and history of medication for diabetes, hypertension and high cholesterol (yes or no).
‡ Incident rate per 1,000 person years.

# Table S13. Hazard ratios (95% CIs) of mortality with the number of weight loss strategies in NHANES 1999-2018 (further adjusted survey rounds and country of birth, N=48,430)

| **Mortality** | **The number of weight loss strategies** | | | | | ***P* value for trend** | **Per 1-strategy increment** |
| --- | --- | --- | --- | --- | --- | --- | --- |
|  | **0** | **1** | **2** | **3-4** | **≥5** |  |  |
| **All cause** |  |  |  |  |  |  |  |
| Incident rate‡ | 19.83 | 14.91 | 12.32 | 10.94 | 5.80 |  |  |
| Cases/N | 5418/28365 | 647/4616 | 576/5043 | 703/6881 | 195/3525 |  |  |
| Model1* | 1.00 (reference) | 0.89 (0.82 to 0.96) | 0.81 (0.75 to 0.89) | 0.80 (0.74 to 0.87) | 0.60 (0.52 to 0.69) | <0.00 | 0.91 (0.89 to 0.93) |
| Model2† | 1.00 (reference) | 0.93 (0.86 to 1.02) | 0.89 (0.81 to 0.97) | 0.89 (0.82 to 0.97) | 0.71 (0.62 to 0.83) | <0.00 | 0.95 (0.92 to 0.97) |
| **CVD** |  |  |  |  |  |  |  |
| Incident rate‡ | 5.02 | 4.12 | 3.34 | 3.08 | 1.07 |  |  |
| Cases/N | 1372/28365 | 179/4616 | 156/5043 | 198/6881 | 36/3525 |  |  |
| Model1* | 1.00 (reference) | 1.00 (0.86 to 1.17) | 0.91 (0.77 to 1.08) | 0.96 (0.82 to 1.11) | 0.49 (0.35 to 0.69) | 0.00 | 0.94 (0.90 to 0.98) |
| Model2† | 1.00 (reference) | 1.00 (0.85 to 1.18) | 0.94 (0.79 to 1.12) | 0.99 (0.85 to 1.17) | 0.55 (0.39 to 0.77) | 0.05 | 0.96 (0.91 to 1.00) |
| **Cancer** |  |  |  |  |  |  |  |
| Incident rate‡ | 4.43 | 3.43 | 2.99 | 2.62 | 1.40 |  |  |
| Cases/N | 1210/28365 | 149/4616 | 140/5043 | 168/6881 | 47/3525 |  |  |
| Model1* | 1.00 (reference) | 0.89 (0.75 to 1.06) | 0.85 (0.72 to 1.02) | 0.83 (0.70 to 0.97) | 0.60 (0.44 to 0.80) | <0.00 | 0.92 (0.88 to 0.96) |
| Model2† | 1.00 (reference) | 0.91 (0.76 to 1.09) | 0.89 (0.74 to 1.07) | 0.87 (0.73 to 1.03) | 0.65 (0.48 to 0.88) | 0.00 | 0.93 (0.89 to 0.98) |

* Model 1 was adjusted for baseline age (years, continuous) and sex (male or female).

† Model 2 was additionally adjusted by race/ethnicity (Hispanic, or non-Hispanic), education level (less than high school, high school or equivalent, or college or above), marital status (married, widowed, divorced, separated, or never married), family income poverty ratio (ratio, continuous), weight change since last year (pounds, continuous), waist circumference (cm, continuous), BMI (normal, overweight, or obesity), self-considered weight status (underweight, about the right weight, or overweight), smoking status (never, past, or current smoker), drinking status (never, past or current), physical activity (yes or no), total energy intakes (kcal/d, continuous), family history of prediabetes or diabetes and heart diseases (yes or no), baseline history of diabetes, hypertension, high cholesterol, coronary heart disease and cancer (yes or no), history of medication for diabetes, hypertension and high cholesterol (yes or no), survey rounds (continuous) and country of' birth (US born, or non-US born).
‡ Incident rate per 1,000 person years.
